# A Fully Integrated Orthodontic Aligner With Force Sensing Ability for Machine Learning‐Assisted Diagnosis

**DOI:** 10.1002/advs.202411187

**Published:** 2024-11-19

**Authors:** Hao Feng, Wenhao Song, Ruyi Li, Linxin Yang, Xiaoxuan Chen, Jiajun Guo, Xuan Liao, Lei Ni, Zhou Zhu, Junyu Chen, Xibo Pei, Yijun Li, Jian Wang

**Affiliations:** ^1^ State Key Laboratory of Oral Diseases National Clinical Research Center for Oral Diseases Department of Prosthodontics West China Hospital of Stomatology State Key Laboratory of Polymer Materials Engineering Polymer Research Institute Sichuan University Chengdu 610041 China; ^2^ Key Laboratory of Testing Technology for Manufacturing Process of Ministry of Education Southwest University of Science and Technology Mianyang 621010 China; ^3^ Tianfu Institute of Research and Innovation Southwest University of Science and Technology Chengdu 610299 China; ^4^ Tianfu Yongxing Laboratory Keyuan S Rd Chengdu 610213 China

**Keywords:** electrospinning, force sensing, malocclusion diagnosis, piezoelectric nanogenerator, wearable devices

## Abstract

Currently, the diagnosis of malocclusion is a highly demanding process involving complicated examinations of the dental occlusion, which increases the demand for innovative tools for occlusal data monitoring. Nevertheless, continuous wireless monitoring within the oral cavity is challenging due to limitations in sampling and device size. In this study, by embedding high‐performance piezoelectric sensors into the occlusal surfaces using flexible printed circuits, a fully integrated, flexible, and self‐contained transparent aligner is developed. This aligner exhibits excellent sensitivity for occlusal force detection, with a broad detection threshold and continuous pressure monitoring ability at eight distinct sites. Integrated with machine learning algorithm, this fully integrated aligner can also identify and track adverse oral habits that can cause/exacerbate malocclusion, such as lip biting, thumb sucking, and teeth grinding. This system achieved 95% accuracy in determining malocclusion types by analyzing occlusal data from over 1400 malocclusion models. This fully‐integrated sensing system, with wireless monitoring and machine learning processing, marks a significant advancement in the development of intraoral wearable sensors. Moreover, it can also facilitate remote orthodontic monitoring and evaluation, offering a new avenue for effective orthodontic care.

## Graphical abstract

The fully integrated occlusal force sensing aligner, constructed using piezoelectric fibres embedded in a flexible printed circuit, demonstrates high sensitivity, a broad measurement range, and reproducible performance in occlusal force monitoring. With the assistance of machine learning, following training on large‐scale clinical samples, it enables the efficient diagnosis of malocclusion and deleterious oral habits.

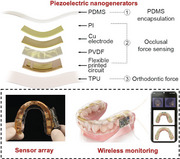

## 1 Introduction

Malocclusion, characterized by the misalignment of teeth and jaws, is a prevalent dental aesthetic disorder that significantly impacts first impressions related to physical attractiveness, career success, intelligence, and overall well‐being.^[^ ^1^, ^2^, ^3^, ^4^ ^]^ Orthodontics is the most effective method for treating malocclusion, and accurate classification of malocclusion is key to successful orthodontic treatment. However, there are several limitations to the clinical classification of malocclusion, including a shortage of orthodontists,^[^ ^5^ ^]^ increased radiation exposure and economic burden on patients due to radiographic examinations,^[^ ^6^ ^]^ and high professional requirements for doctors, which pose a risk of misdiagnosis.^[^ ^7^ ^]^ Therefore, there is an urgent need for the development of more efficient and non‐radioactive tools for the classification of malocclusion.

Dental occlusion provides critical insights into the functional status of tooth arrangement and temporomandibular muscles, which vary significantly across different types of malocclusions, making them essential factors for the classification of different malocclusion types.^[^ ^8^, ^9^, ^10^ ^]^ The current occlusion monitoring technologies have various limitations, such as inflexible designs, high power requirements, and limited capabilities, which may lead to daily discomfort and restricted medical application.^[^ ^11^ ^]^ Numerous research groups have attempted to develop effective and flexible occlusal pressure sensors based on piezoresistive,^[^ ^12^ ^]^ capacitive,^[^ ^13^ ^]^ electromagnetic,^[^ ^14^ ^]^ and optical transduction mechanisms.^[^ ^15^ ^]^ However, despite their ability to accurately detect physiological signals generated by bite force, the dependence of these pressure sensors on a voltage supply presents a significant barrier to their miniaturization and seamless integration. Piezoelectric nanogenerators (PENGs) have significant application potential as wearable power sources and self‐powered sensing technologies, owing to their superior mechanical‐to‐electrical energy conversion efficiency.^[^ ^16^, ^17^, ^18^, ^19^, ^20^ ^]^ However, the development of a fully integrated, flexible, PENG‐based bite force‐sensing device has not yet been reported. Moreover, PENG‐based sensors face several challenges in signal processing, such as signal noise, poor stability, and low reliability, limiting their application in the medical field.

Machine learning (ML)‐based sensor data analysis provides a new strategy to overcome the challenges of flexible electronic sensors.^[^ ^21^, ^22^, ^23^ ^]^ ML enhances the effectiveness of flexible electronic sensors by processing complex data, analyzing noisy inputs, identifying hidden relationships, and mining interrelations between signals and biological events.^[^ ^24^ ^]^ Integrating wearable electronic devices with ML algorithms allows for comprehensive analysis and data mining of physiological signals, such as heart rate,^[^ ^25^ ^]^ blood pressure,^[^ ^26^ ^]^ blood oxygen saturation,^[^ ^27^ ^]^ laryngeal activities,^[^ ^28^ ^]^ and biochemical markers.^[^ ^29^ ^]^ This approach significantly enhances the capabilities of flexible electronic technology in disease diagnosis and precision treatment. However, preliminary testing of these wearable devices with fewer subjects may lead to less accurate model predictions during their clinical application on new individuals. Therefore, the size of the test subject population, and not the ML model algorithm, is the key factor affecting the practicality and application of flexible electronic sensors for ML‐assisted health monitoring.

In this study, we introduced a fully integrated artificial intelligence‐enhanced invisible aligner (ARIA) equipped with powerful long‐term sensing capabilities for monitoring occlusal force and aiding in malocclusion diagnosis. ARIA is constructed from a nano‐confined, electrospun nanofiber (NCEN)‐based PENG utilizing polyvinylidene fluoride–trifluoro‐ethylene (PVDF‐TrFE/MXene) nanocomposites, and it can efficiently convert biomechanical energy from teeth percussion into electrical energy. The ARIA system integrates the NCEN‐PENG with miniaturized wireless control electronic systems into a soft and wearable format that can overcome the aforementioned limitations. ARIA can process multiple signal channels from specific teeth positions in real‐time, condition these signals on‐board, and wirelessly transmit them to a mobile terminal with high precision. In the terminal, innovative software has been developed, integrating clinical samples from over 1400 individuals and multiple ML algorithms, to proficiently transform sensor signals into virtual teeth models for precise malocclusion classification. The fully integrated ARIA system reduces orthodontists’ reliance on traditional radiographic examinations and medical expertise, thus broadening its accessibility and applicability in clinic and daily life settings.

## 2 Results and Discussion

### 2.1 Design of ARIA

**Figure 1:**
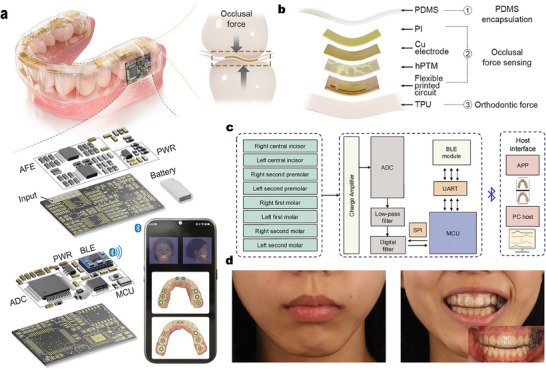
Overview of the fully integrated ARIA system a) Schematic diagram of ARIA featuring the piezoelectric sensor array on the occlusal surface of the teeth, printed circuit board, and battery. The exploded view of the board (bottom left) shows the analog front‐end, analog‐to‐digital converter, and microcontroller unit components. b) Structural scheme of a 6‐layer composite flexible piezoelectric sensor. c) A block diagram showing the workflow of ARIA, including signal processing, control, communication, and display. d) Closed‐mouth and smiling photographs of a subject wearing ARIA. The inset displays an intraoral photo of the maxillary teeth fitted with ARIA.

ARIA is composed of hardware with wireless communication capabilities and software based on sophisticated ML algorithms. The hardware component first collects and transmits the bite force signals, which are then comprehensively analyzed by the cloud‐based algorithms. The hardware in ARIA is composed of nanofiber‐based flexible NCEN‐PENG sensor arrays and control electronics, which are encapsulated in a miniaturized and soft format (**Figure** **1**a). The flexible NCEN‐PENG sensor arrays consist of a hollow PVDF‐TrFE/MXene electrospun nanofiber membrane (hPTM‐ENMs) and a flexible printed circuit (FPC). The NCEN‐PENG sensor matrix is distributed on the occlusal surface of the left central incisor (LCI), right central incisor (RCI), left first premolar (LFP), right first premolar(RFP), left first molar(LFM), right first molar(RFM), left second molar(LSM), and right second molar(RSM). The control electronics are designed on a printed circuit board (PCB) for occlusal sensing and wireless communication. The circuitry consists of an analog front‐end (AFE) and a data acquisition (DAQ) module. ARIA, produced through a cost‐effective process, exhibits exceptional mechanical stability under various deformations, such as squeezing and twisting (Figure S1, Supporting Information). Intraoral bite force sensing requires careful consideration of sensor size, as it is crucial for both wearing comfort and the accuracy of bite force measurement. The six‐layer structure of ARIA is composed of an 8‐pixel NCEN‐PENG‐based sensor array and PCB (Figure 1b). The bottom layer consists of a thermoplastic polyurethane‐based orthodontic appliance; the middle layer comprises the FPC, which includes the NCEN‐PENG sensor matrix and PCB; and the top layer consists of the polydimethylsiloxane packaging layer. The packaging mold, with a 0.3‐mm upper layer, was designed using 3D printing technology to ensure conformation to the dentition shape and effective mechanical conduction (Figure S2, Supporting Information). This flexible packaging on the occlusal surface guarantees efficient transmission of bite force. Several NCEN‐PENG‐based sensor units were integrated into an array to enable multi‐channel measurement, which supports self‐powered bite force imaging. Deformation of the NCEN‐PENG sensor array leads to the transmission of the 3D occlusal information, including occlusal position, force, and contact timing, of the entire dentition to the acquisition circuit. After signal processing, including signal amplification, filtering, and separation, the data is displayed on computer and mobile phone interfaces via Bluetooth (Figure 1c). After integration, the invisible aligner maintains its aesthetic and transparent benefits (Figure 1d). Therefore, ARIA can distinguish between various adverse oral habits and bite patterns of malocclusion with efficient ML algorithms, facilitating remote monitoring and orthodontic effect evaluation.

### 2.2 Characteristics and Working Mechanism of the NCEN‐PENG Sensor

**Figure 2:**
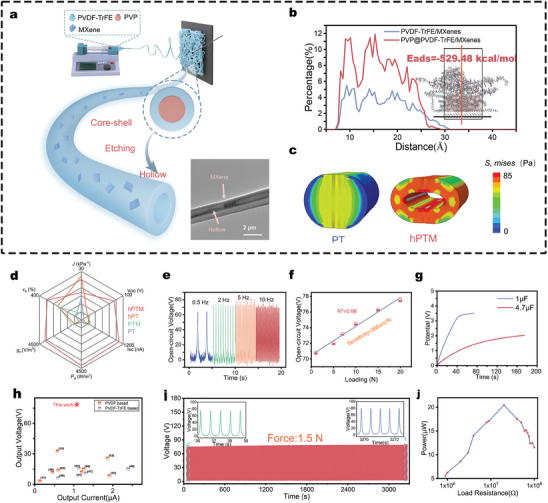
Design and characterization of high‐performance piezoelectric sensors a) Schematic representation of the preparation of the hPTM fibers and its TEM image showing MXene nanosheets embedded in the PVDF matrix (scale bar: 2 µm). b) The fluorine atom concentration distribution of PVDF‐TrFE at 3 ns from MD simulations is depicted, with the red curve representing the results after the introduction of PVP. The inset shows the distribution of PVDF‐TrFE molecular chains at this time, with the red regions indicating PVDF‐TrFE, and PVP located above these regions. c) Results of FE analysis of the deformation of PVDF‐TrFE and hPTM fibers under the pressure applied by two parallel plates. d) Comparison of piezoelectric fibrous membranes across six performance metrics: elongation at break, flexibility, output voltage, output current, volume charge coefficient, and piezoelectric power density. The red sections represent the performance of hPTM‐ENMs. e,f) Open‐circuit voltage of hPTM‐ENMs at different frequencies e) and loads f). g) Charge voltage diagrams of electronics for 0.1 and 4.7 µF capacitors. h) Comparison of the piezoelectric output of hPTM‐ENMs with previously developed PENGs. i) Long‐term stability testing of hPTM‐ENMs. j) Power variations of hPTM‐ENMs at increasing load resistance.

The hPTM‐ENM, a core component of the NCEN‐PENG sensor array, is fabricated through electrospinning (**Figure** **2**a). The synergistic effect of MXene doping and polyvinylpyrrolidone (PVP) polymer core‐shell confinement during the spinning process results in the formation of nanofibers with exceptionally high β‐phase content.^[^ ^30^ ^]^ The presence of hollow channels facilitates greater strain under tooth movement‐associated mechanical force,^[^ ^31^ ^]^ enabling the hPTM‐ENMs to produce extremely high and sensitive piezoelectric outputs.^[^ ^32^, ^33^, ^34^ ^]^ Furthermore, MXene's electromagnetic shielding minimizes the effects of electromagnetic waves generated inside the sensor on living organisms.^[^ ^35^ ^]^ Detailed analyses of the nanofibers' morphology, phase transitions, and structural design are presented in Note S1, with illustrations in Figures S3 and S4 (Supporting Information).

Two models were constructed and studied using Molecular Dynamics simulation (MDs) software and Finite Element (FE) analysis software to determine the mechanisms underlying the significant phase transformations driven by synergistic effects in the systems containing MXene, PVDF‐TrFE, and PVP. The results of MDs demonstrate that the strong electrostatic interactions between MXene and macromolecular chains drive the self‐assembly ^[^ ^36^, ^37^ ^]^ of the hPTM‐ENMs. The addition of PVP molecular chains above the PVDF‐TrFE and MXene interaction space, leads to a notable increase in the relative concentration of fluorine atoms,^[^ ^38^, ^39^ ^]^ perpendicular to the MXene substrate. This compression of the fluoropolymer chains (Figure 2b) confirms that PVP confinement enhances the MXene‐induced dipole polarization ^[^ ^32^, ^33^ ^]^ of PVDF‐TrFE. Therefore, MDs demonstrates one of the mechanisms underlying the synergistic effects of MXene, PVDF‐TrFE, and PVP. The MDs process and comparative analysis of PVDF–TrFE polarization, before and after PVP confinement, are detailed in Note S2 and Figure S5 (Supporting Information).

The piezoelectric and ferroelectric properties of the hPTM‐ENMs were analyzed and studied using piezoresponse force microscopy (PFM) (Figure S6, Supporting Information). The results of PFM (Note S3, Supporting Information) and MDs confirm the critical role of synergistic effects during the electrospinning process on the ferroelectric properties of the hPTM‐ENMs,^[^ ^34^ ^]^ which enables them to produce exceptionally high piezoelectric outputs without relying on electric field polarization.^[^ ^32^ ^]^

ENMs capable of greater displacement can produce higher piezoelectric outputs under the same applied pressure.^[^ ^31^ ^]^ FE analysis was used to study the changes in fiber modulus before and after the addition of MXene and the strain produced by the fibers under compression with and without the presence of hollow channels (Figure S7, Supporting Information). The effective strain in the fibers decreased with an increase in the PVDF‐TrFE modulus. Additionally, the introduction of hollow channels ^[^ ^40^ ^]^ in the hPTM can offset the reduction in effective strain caused by MXene doping (Figure 2c). Therefore, despite the mechanical weakening caused by the formation of hollow channels, the inclusion of MXene endowed the structurally limited and mechanically weaker PVDF‐TrFE ENMs with considerable tensile strength and toughness (Figure S4e, Supporting Information), while maintaining its applicability in the oral environment.

The MDs and FE analysis revealed that MXene effectively induces polarization of PVDF‐TrFE during the electrospinning process,^[^ ^41^ ^]^ which is further enhanced by PVP confinement, resulting in core‐shell structured ENMs with high piezoelectric responsiveness.^[^ ^42^ ^]^ Additionally, the PVP solution, located in the core layer, is etched to form hollow channels that contribute to greater effective strain.^[^ ^31^ ^]^

The hPTM‐ENMs exhibited the highest open‐circuit voltage and short‐circuit current during periodic impact tests (Figure S8, Supporting Information), reaching 78.5 V and 1.08 µA, respectively, representing a 13.3‐fold and 12.5‐fold increase over the original core‐shell PVDF‐TrFE ENMs. Evidently, the introduction of MXene or hollow structures alone is insufficient to significantly enhance the piezoelectric output,^[^ ^32^, ^43^ ^]^ as their synergistic effect enables the substantial increase in the piezoelectric output of the electronic devices. Figure 2d provides a visual comparison of the various performance metrics of PVDF‐TrFE before and after the introduction of a hollow structure and MXene fillers. The hPTM‐ENMs exhibit superior performance across six metrics: elongation at break, flexibility, output voltage, output current, volume charge coefficient, and piezoelectric power density. Consequently, under the influence of the synergistic effects of hollow structures and MXene fillers, hPTM‐ENMs can exhibit superior mechanical properties while delivering high piezoelectric output, compared to the other three samples.

Moreover, hPTM‐ENMs demonstrated a highly stable and reproducible signal output at various frequencies (0.5, 2, 5, and 10 Hz) (Figure 2e). Within 0.5‐20 N impact force range, the slope of the open‐circuit voltage with applied load (defined as force sensitivity) reached 366 mV N^−1^ (Figure 2f), which is significantly higher than the 18.45 mV kPa^−1^ reported in another study ^[^ ^33^ ^]^ on piezoelectric composite fiber membrane strain sensor (Table S1, Supporting Information). These reports confirm the sensitivity of the piezoelectric response of hPTM‐ENMs due to the high β crystal content.

The piezoelectric energy generated by the hPTM‐ENMs, in the form of alternating current output signals, was converted into direct current signals through a rectifier bridge ^[^ ^44^ ^]^ and stored in capacitors of 1 and 4.7 µF during periodic impact experiments. The piezoelectric device could charge a 1 µF capacitor to 3.39 V within 50 s and a 4.7 µF capacitor in 180 s (Figure 2g). Furthermore, comparative analysis of the piezoelectric performance of various PVDF‐based and PVDF‐TrFE‐based nanogenerators (Figure 2h) revealed that the output voltage of hPTM‐ENMs is significantly higher than that of the other PENGs at a moderate output current, indicating its superior piezoelectric output.^[^ ^31^, ^33^, ^41^, ^45^, ^46^, ^47^, ^48^, ^49^, ^50^, ^51^, ^52^, ^53^ ^]^ Furthermore, long‐term fatigue tests on the piezoelectric device revealed that the output electrical signal showed no significant fluctuation even after > 3000 cycles (1.5 N, 2 Hz), confirming their high stability (Figure 2i). Figure 2j illustrates the power variation of hPTM‐ENMs as the load impedance changes from 0.9 to 100 MΩ, which allows for an assessment of their output performance. The power of hPTM‐ENMs initially increases and then decreases with an increase in load resistance, reaching a peak (20.45 µW) at 20 MΩ load resistance. The studies on the piezoelectric and mechanical properties of hPTM–ENMs demonstrate their capability and reliability for operating within the oral environment.

### 2.3 Monitoring System of ARIA

The monitoring system for human occlusion was designed to quantitatively analyze the distribution of bite force within a designated area of the aligner. The AFE achieves occlusion sensing through coordinated sequence control of multiple components. However, considering the large output impedance of the piezoelectric film, the FPC with the integrated sensor array cannot be directly connected to the DAQ module, as the corrected voltage signal will not be measurable (Note S3, Supporting Information). Therefore, an impedance‐matching circuit was designed at the front end of signal acquisition. Additionally, the input impedance of the measurement system was improved by using an operational amplifier as a buffer in a voltage follower. The circuit PCB features a 6‐layer board design, consisting of a microcontroller unit (MCU), analog‐to‐digital converter (ADC), and Bluetooth module on the top layer and charge amplifier on the bottom layer. This arrangement effectively reduces interference from the digital circuit on the analog circuit (Figure S9, Supporting Information). Additionally, the key signal lines are routed in the middle layers, enhancing the electromagnetic compatibility of the entire system. Moreover, owing to its small size, the PCB (30 mm × 20 mm) can be easily packed into the aligner without causing noticeable discomfort to the user (Figure S10, Supporting Information).

When subjected to occlusal pressure, the NCEN‐PENG sensor generates an alternating current signal. The negative voltage of the signal is detected by a voltage shifter, designed using an additional circuit composed of an operational amplifier, which further improves signal stability and signal‐to‐noise ratio. Thereafter, the ADC uses Analog Devices’ AD7606 to achieve synchronous sampling of eight sensor output signals. This chip offers 16‐bit, 8‐channel synchronous sampling, with a maximum sampling rate of 200 kSPS and integrates a voltage reference source and a digital resistor. Additionally, its anti‐aliasing filter enables ± 5 V bipolar signal processing with a 5 V single power supply. These characteristics of the AD7606 simplify its peripheral circuit design and ensure compatibility with the mainstream 3.3 V communication level of the processor. Moreover, since AD7606 incorporates a built‐in low‐pass filter, no additional filter circuit is necessary to eliminate frequencies above 20 Hz, thereby simplifying the circuit design. AD7606 supports both parallel and serial data transmission. The MCU generates a PWM signal as the conversion signal for the AD7606 through an internal timer to manage the sampling rate. The MCU and ADC communicate via the serial peripheral interface, and the MCU sends a clock signal to control data transmission. To handle data storage at higher sampling rates, the ADC output signal is first temporarily stored in the MCU's static random access memory. After complete data collection, the data is sent to the Bluetooth module and transmitted to a terminal device (such as a computer or a smartphone) via Bluetooth. The Bluetooth module can transmit wide‐band signals at a distance of ≈20 m at 4.0 Mbps with zero data loss (Figure S11, Supporting Information). The ARIA monitoring system has a standby current of ≈12 mA and a working current of ≈53 mA during sampling.

Ensuring the independence of each sensor in the array or each channel in the system is vital to minimize mutual crosstalk. Analysis of the typical electrical signals of three adjacent sensors (LCI, RCI, and LFP) by Oscilloscope revealed that when pressed, the electrical output signals of LCI were significantly higher than those of the RCI and LFP, indicating minimal signal interference. The sensor had a response time (voltage rising from 10% to 90% of peak voltage) and hysteresis time (voltage dropping from 90% to 10% of peak voltage) of 42 and 26 ms, demonstrating its rapid response characteristics (Figure S12, Supporting Information), which ensures that the sensor can promptly detect signals when an external force is applied.

### 2.4 Monitoring of Various Occlusions in the Oral Cavity

**Figure 3:**
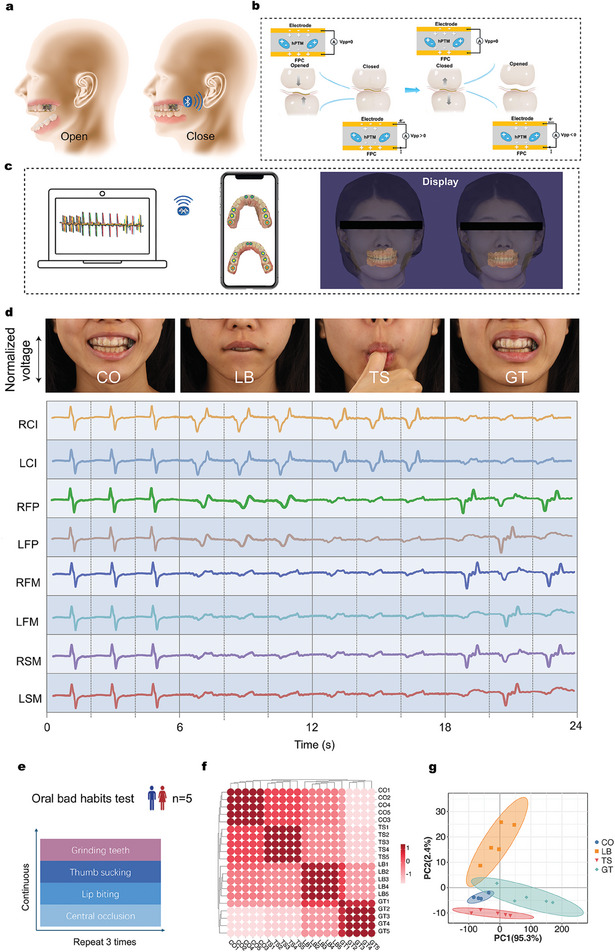
Evaluating ARIA for monitoring occlusal force and bad oral habits during daily activities. a) Schematic diagram of the open and closed mouth fitted with ARIA. b) Schematic diagram of the four stages of charge transfer during the tooth contact‐separation cycle of an occlusal bite. c) The ARIA signals can be transferred to PC terminals and smartphones via Bluetooth, which then process and display them on an interactive interface. d) Photographs of the subjects wearing ARIA and performing CO, LB, TS, and GT, and the corresponding voltage curve generated during these activities. e) Control experiment conducted using five healthy subjects to assess the reliability of ARIA in monitoring bad oral habits. f) Correlation heatmap of data from five subjects testing CO, LB, TS, and GT occlusion conditions. g) Cluster analysis of data obtained from five subjects testing CO, LB, TS, and GT after PCA.

**Figure** **3**a,b illustrate the motion cycle of the NCEN‐PENG within ARIA during the routine occlusal process. The peak‐to‐peak voltage in an open circuit between the bottom electrode of the FPC (V_FPC_) and the top copper (Cu) electrode (V_Cu_) is defined as V_pp_ (V_pp_ = V_Cu_ − V_FPC_). In the initial occlusal opening stage, the stored charge pairs and the induced opposite charges on the Cu electrodes are in electrostatic equilibrium, resulting in no voltage difference between the FPC and Cu electrode (V_pp_ = 0). However, as the occlusal closing phase progresses, the hollow microstructures within the hPTM‐ENMs deform, causing the electrodes to move closer together. This reduces the overall thickness of the piezoelectric layer, thereby decreasing the polarization intensity of the macroscopic dipoles and inducing electron flow from the Cu electrode to FPC through the hPTM‐ENMs (V_pp_ > 0). At the fully closed occlusal stage, the thickness of the piezoelectric layer reaches its minimum, achieving maximum charge transfer and establishing a new electrostatic equilibrium for the NCEN‐PENG (V_pp_ = 0). In the subsequent occlusal opening phase, the NCEN‐PENG returns to its elastic state (V_pp_ < 0), and the pressure is gradually relieved, allowing the deformed microstructures to revert to their original shape, resulting in the flow of electrons from the FPC back to the Cu electrode until the teeth return to the initial opened stage. This contact‐separation working principle effectively generates discrete pulsed electrical fields.

Occlusal status offers rich mechanical and distributional information for the clinical diagnosis of various oral diseases and post‐treatment evaluations. Therefore, it is crucial to measure the occlusion status of orthodontic patients to determine the treatment prognosis and oral health (teeth, periodontal ligaments, and masticatory muscle status) for the early prevention of malocclusions caused by bad dental habits. The aforementioned NCEN‐PENG pressure sensor can accurately identify stress generated by different frequencies in the same site or constant frequency in different sites, as well as different stresses at the same site, reflecting the oral health status during biting. The NCEN‐PENG sensor in the ARIA system has a sampling frequency of ≤ 50 Hz, enabling it to continuously monitor changes in the occlusal state. This system can measure the occlusal data of eight different sites, allowing the integrated system to monitor and distinguish various occlusal states, such as centric occlusion (CO), lateral occlusion, and bad oral habits.

The ARIA system was used to analyze various movements of the stomatognathic system using its 8‐site sensor array. When multiple teeth make contact simultaneously, the occlusal force information from each tooth is recorded and synthesized into a complex electrical signal corresponding to a unique occlusion relationship. Although the data collection channels for the eight tooth sites function independently, their integration allows the system to comprehensively monitor the movement of the stomatognathic system. When occluding in centric relation, the integrated sensors within the system are compressed, promptly converting the mechanical pressure into electrical signals, which are then dynamically displayed in real‐time on the dental model in a smartphone (Figure 3c and Movie S1, Supporting Information). During lateral chewing movements, pressure is localized to one side of the tooth, and this asymmetry is distinctly shown on the display model (Movie S2, Supporting Information). This responsiveness of the ARIA system allows it to adapt instantly to substantial changes in the occlusal state, thereby providing an accurate and immediate pressure map of the tooth's occlusal surface. The low power consumption and sustainable detection capabilities of ARIA allow it to monitor bad oral habits, such as lip biting (LB), thumb sucking (TS), and grinding teeth (GT), in addition to detecting the bite status of teeth (Figure 3d; Movies S3 and S4, Supporting Information). Current research on the pathogenic mechanisms of adverse oral habits indicates that the abnormal pressures resulting from these habits can disrupt the muscle force balance within and outside the oral and maxillofacial system during growth and development. This disruption can lead to maldevelopment and morphological anomalies of the teeth, dental arches, and jawbones, resulting in varying degrees of malocclusion.^[^ ^54^ ^]^ Thus, early identification of detrimental oral habits and implementation of interceptive therapy are essential for preventing malocclusion.^[^ ^55^ ^]^

A control experiment was conducted on five healthy subjects to assess the reliability of ARIA in monitoring bad oral habits (Figure 3e). The subjects were fitted with ARIA and asked to perform various activities, including CO, LB, TS, and GT, during which the dynamic curves from all the individual sensors were obtained (n = 20 datasets). Our observations indicated that in CO, all teeth experienced pressure and produced output voltage signals. Meanwhile, during LB or TS, occlusal pressure signals were detected only in the anterior teeth and some posterior teeth. Additionally, during GT movements, the occlusal force signals in the posterior teeth alternated between the left and right sides. Pearson correlation analysis (PCA) of the monitoring data revealed that the characteristics of bad oral habits were highly consistent, while the characteristics of different occlusal states showed clear distinctions across different subjects (Figure 3f). Furthermore, PCA dimensionality reduction analysis revealed that different occlusal states can be clearly distinguished between different subjects (Figure 3g).

### 2.5 Application in the Classification of Malocclusion

**Figure 4:**
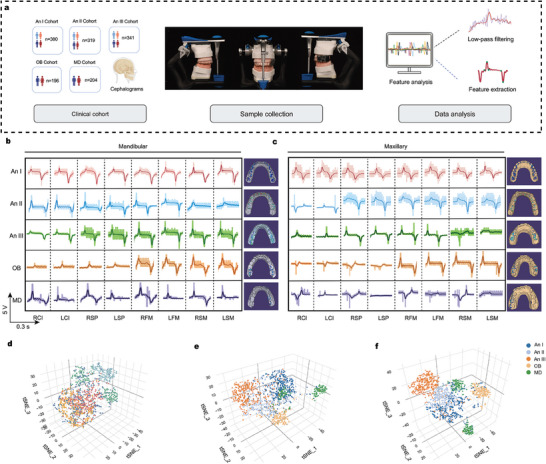
Utilizing ARIA to collect occlusal data from various malocclusions in a large clinical sample a) Overview of employing ARIA for the collection of 1467 clinical samples and extraction of comprehensive data. b,c) Sensor signals were acquired from five different malocclusion models wearing ARIA on the maxilla b) and mandible c). d,e) t‐SNE plots showing mandibular d) and maxillary e) feature separation in a 3D space. f) Feature vector matrices of the maxillary and mandibular structures simultaneously integrated using the t‐SNE algorithm.

The classification of malocclusion plays a significant role in devising future orthodontic treatment strategies, as well as influencing the progression of orthodontic discipline. An ideal classification of orthodontic conditions could summarize the diagnostic data (e.g., dental casts, radiographs, and photographs) and suggest corresponding treatment strategies. Although there is no optimal approach to categorizing malocclusions, Angle's classification is the most universally adopted and recognized method. In Angle's classification, based on the sagittal relationship and tooth arrangement of the upper and lower first molars, malocclusions are classified into three categories: An I, An II, and An III malocclusions. However, recent research has indicated that it is essential to consider other characteristics, such as transverse relationships (e.g., mandibular deviation) and vertical relationships (e.g., open bite, OB) for a comprehensive classification of malocclusions.^[^ ^56^ ^]^ To assess the application of ARIA in malocclusion classification, we retrospectively analyzed a comprehensive dataset of 1467 patient models from individuals seeking orthodontic treatment at the West China Hospital of Stomatology (**Figure** **4**a). Each participant included in the study underwent a cephalometric examination and dental cast recording. Malocclusion diagnosis of these patients was performed independently by two experienced orthodontists.

The occlusal movement of teeth is a complex process involving the coordination of masticatory muscles and temporomandibular joint. Consequently, using an external device is essential to accurately restore the true occlusion of the patient. Mechanical articulators can accurately simulate the position of the condyle and mandibular movements, making them widely popular in orthodontics, oral rehabilitation, and temporomandibular joint treatment.^[^ ^57^ ^]^ In this study, 1467 plaster casts were mounted on fully adjustable articulators to mimic the intra‐oral occlusion of the patients (Figure S13, Supporting Information). Thereafter, dynamic data from all the integrated sensors in the ARIA were collected while mimicking CO (Figure 4b). Angle I malocclusion shows no abnormal sagittal arrangement but may involve dental crowding and overbite, which is reflected in the sampling signal with stable voltage signals across all teeth. In Angle II malocclusion, the mandibular second molar signal is smaller than the maxillary second molar signal, and the upper anterior teeth signals are smaller than the lower anterior teeth signals. Lastly, in Angle III malocclusion, the maxillary second molar signal is weaker than the mandibular second molar signal and the lower anterior teeth signals are smaller than the upper anterior teeth signals. Abnormal tooth arrangements in vertical and horizontal directions also exhibit distinct voltage signal patterns. For example, the OB model shows a unique electrical signal pattern, consisting of a simultaneous decrease in the signal intensity of the upper and lower anterior teeth sites, but a stable signal intensity of the posterior teeth site. Meanwhile, the mandibular deviation model shows an asymmetric voltage signal distribution between the left and right sides. These signal characteristics align with the clinical manifestations of malocclusion.

To further verify the reliability of ARIA in classifying malocclusions, we extracted features from each valid waveform data, selecting the maximum value, minimum value, peak‐to‐valley interval, number of zero crossings, number of inflection points, and absolute square value as eigenvalues. These extracted eigenvalues were combined into a data matrix, and t‐distributed stochastic neighbor embedding was performed to project and display all the malocclusion data. When data from only the eight mandibular channels were included, the discrimination among the five classifications was not very clear (Figure 4d), and although the maxillary data showed better discrimination than the mandibular data, it was not ideal (Figure 4e). However, after including data from both the upper and lower jaws and 16 channels simultaneously, the five malocclusions were well distinguished in the 3D scale (Figure 4f).

### 2.6 ML Model for Malocclusion Classification

**Figure 5:**
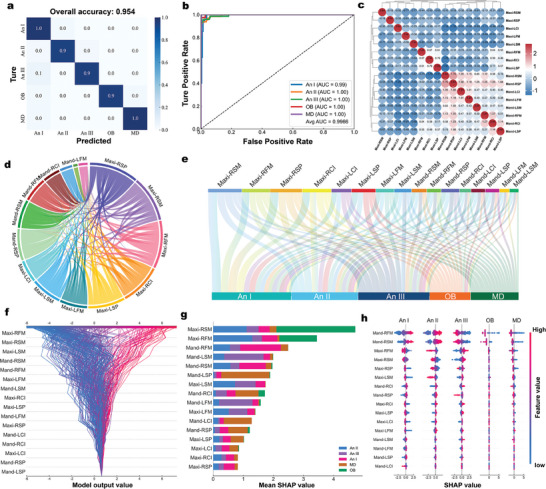
Classification of malocclusions based on ML a) Confusion matrix showing the accuracy of the XGBoost ML model in predicting the malocclusion in the test set. b) ROC curve of the XGBoost ML model for predicting five types of malocclusions. c) Correlation heatmap between 16 features of maxilla and mandible. d) Chord diagram showing the relative correlation between different teeth. e) Sankey diagram of SHAP analysis depicting the relative contribution of different teeth to malocclusion classification. f) SHAP decision plot of the ML model predicting different malocclusions using maxillary and mandibular data. g) Stacked bar plot of feature importance showing the contribution of each tooth to each malocclusion type. h) SHAP summary plot of the XGBoost ML model based on the dataset collected by ARIA.

ML presents a distinct advantage for the efficient analysis and processing of piezoelectric signals, as ML can detect even small differences in signals that are often neglected by humans.^[^ ^58^, ^59^, ^60^ ^]^ Seven ML algorithms were employed to classify the voltage signals generated by tooth‐surface contact when the lower teeth (mandible) and upper teeth (maxilla) were in the central position to enhance the accuracy and robustness of the detection system. Among the evaluated ML models, the Extreme Gradient Boosting (XGBoost) boosted decision tree model outperforming typical ML models, such as Back Propagation (BP) Neural Networks, Support Vector Machines (SVM), Linear Regression, Random Forest, K‐Nearest Neighbors (KNN), and Decision Trees (Figures S14–S20, Note S5 and Table S3, Supporting Information). By integrating sensor data from both the upper and lower jaws, most models could distinguish the majority of malocclusions with over 90% accuracy. For instance, BP Neural Network achieved an overall accuracy of 93.4% for malocclusion classification, as verified by the normalized loss of the two objective functions during 100 iterations (Figure S14, Supporting Information). However, the XGBoost ML model achieved an overall accuracy of > 95% for malocclusion classification. These findings illustrate the changes in model accuracy with increasing sample size and number of channels. The receiver operating characteristic (ROC) curve is a composite indicator that reflects a continuous variable of sensitivity and specificity, with the vertical coordinate reflecting the true positive rate. The ROC curve demonstrated that the area under the curve of the XGBoost ML model for the five malocclusions was above 0.99, indicating its reliability (**Figure** **5**a,b). **Table** **1** shows the performance of seven commonly used ML models with maxillary, mandibular, and maxillomandibular data. The precision‐recall curve illustrates the performance of various ML models across different data sets. The XGboost and Random Forest ML models show strong performance on the maxillary, mandibular, and combined maxillomandibular data sets (Figures S21–S23, Supporting Information).

**Table 1:** Comparison of the performance of ML models.

ML algorithm	Maxilla	Mandible	Maxilla and mandible
XGboost	0.7951	0.8125	0.9549
SVM	0.8056	0.7812	0.9479
Random Forest	0.7778	0.8194	0.9549
BP neural network	0.6458	0.7535	0.9340
MLP	0.7431	0.6007	0.8924
KNN	0.7396	0.7083	0.9028
Decision Tree	0.6944	0.7396	0.8229

ARIA leverages signal features from 16 specific tooth positions to achieve high‐accuracy classification of malocclusions. However, understanding the correlations between signals from different tooth positions and the contribution weight of each signal to classification decisions is clinically valuable for diagnostic analysis. The Pearson correlation coefficient between all sensors in ARIA highlights the correlation between different tooth positions in malocclusion classification (Figure 5c). The relatively homogeneous correlation suggests that the extracted features are highly independent (Figure 5d). To assess the contribution of each tooth position to the classification model, Shapley additive interpretation (SHAP) was utilized to evaluate the feature importance of 16 tooth positions in the maxillary and mandibular for each malocclusion classification (Figure 5e). SHAP analysis revealed that the relative relationship between the maxillary and mandibular molars is essential for classifying An II, An III, and OB, while the relative relationship of the premolars is critical for classifying MD (Figure 5f–h; Figure S24, Supporting Information). In Angle's malocclusion classification system, the key evaluation criterion is the relative positional relationship between the first molars in the sagittal direction, specifically between the mesiobuccal cusp of the maxillary first molar and the mesiobuccal groove of the mandibular first molar. Currently, there is no classification system for malocclusion that is based on the full spectrum of occlusal information for the entire dentition, including position, size, and the sequence of tooth contact, primarily because obtaining such detailed occlusal information is exceedingly difficult. However, this method cannot determine the magnitude of bite force or the timing of tooth contact. Similar to Angle's classification, SHAP analysis showed that the relative position of molar occlusal information remains the most critical factor in malocclusion classification. The XGBoost model has greatly improved the accuracy of malocclusion classification based on 3D data, even surpassing the accuracy of less experienced orthodontists.

## 3 Conclusion

In this study, we introduced a fully integrated, occlusal force‐sensing, invisible aligner for wireless monitoring and ML‐based analysis of various dental occlusal activities directly from the occlusal surface. This innovative system integrates highly sensitive and flexible piezoelectric sensors that can independently and remotely detect the oral occlusal state and transmit the data to a terminal device via Bluetooth. The aligner continuously monitors the occlusal status and bad oral habits that affect orthodontic results and displays this information on a smartphone terminal to remind patients to correct the bad oral habits and improve the effectiveness of orthodontic treatment. Additionally, the ML model, trained with 1400 cases, can further analyze the bite sensor data to identify the malocclusion type with over 95% accuracy and evaluate the progress of orthodontic treatment. By processing data in the cloud, this device provides new opportunities for remote diagnosis and treatment evaluation in orthodontics.

## 4 Experimental Section

### Fabrication of h‐PVDF‐TrFE/MXene ENMs

A mass of 0.0075 g of MXene powder was admixed with 10 ml of a DMF and Acetone mixture, maintaining a volume ratio of 3:2 between DMF and acetone. This mixture was then sonicated at room temperature for 2 h. Post sonication, 1.5 g of PVDF‐TrFE powder and 0.8 g of PVP powder were incorporated, and the resultant mixture was stirred at 60 °C for 2 h to ensure complete dissolution of the polymer powders. The resulting solution, rich in PVDF‐TrFE, was set aside as the precursor for the outer layer spinning solution. Simultaneously, PVP (K90) powder was dissolved in anhydrous ethanol, stirred at room temperature for 2 h, to prepare a 10% (by mass) inner layer spinning solution. The electrospinning process was conducted using a 21/16G stainless steel coaxial needle, with high direct current voltages of 18 kV and −1.5 kV applied to the needle and the collector electrode, respectively. The feed rates for the inner and outer layer solutions were meticulously controlled at 0.2 and 0.6 mL h^−1^, respectively. The composite fibers were harvested using a collector wrapped in aluminum foil, rotating at a speed of 30 rpm. Subsequently, the composite fiber film was subjected to ultrasound in ethanol for 2 h to extricate the internal PVP, followed by a drying phase in a vacuum oven at 60 °C for 2 h, culminating in the formation of hollow PVDF‐TrFE/MXene composite textiles. For comparison, pure PVDF‐TrFE solutions and PVDF‐TrFE/MXene solutions, devoid of PVP, were directly electrospun using a 21G needle, with a feed rate of 0.2 mL h^−1^. The hollow fibers of pure PVDF‐TrFE were synthesized employing the aforementioned methodology.

Copper foil electrodes were affixed to the upper and lower surfaces of the fiber membrane, with an effective area of 2 cm × 2 cm. The device was encapsulated with PDMS to shield the fiber membrane from environmental influences while ensuring close contact between the copper foil electrodes and the fiber membrane. The mass ratio of PDMS to curing agent was 10:1, and it was cured in an oven at 80 °C for 1 h, ultimately producing h‐PVDF‐TrFE/MXene ENMs.

### ARIA Fabrication

As shown in the Figure 1b, ARIA was composed of an inner layer of transparent aligners, middle hPTM‐ENMs composite textiles and outer layer of PDMS. The manufacturing of ARIA mainly includes i) preparation of invisible aligners, ii) connection of sensors and welding of components, and iii) packaging using PDMS. Participants' oral data were precisely captured with a 3Shape scanning device as detailed in the Figure S25 (Supporting Information). This scan facilitated the creation of tailored plans for the incremental movement of target teeth, reflecting each individual's specific dental needs, which were subsequently exported in STL format. The STL profiles were loaded into Photon Workshop, where the dental models were fabricated using a 3D printer. The thermoplastic urethanes diaphragm was heated to 200 °C, inducing a pressure of 400 kPa on its upper surface, compelling the splint to accurately mimic the rigid tooth model's shape. Excess material was removed to finalize the aligner. Next, eight square copper electrodes and hPTM‐ENMs composite textiles were uniformly positioned at designated locations on a dental arch‐shaped FPC. The size of each electrode and nanomembrane is 3*5 mm (anterior teeth), 5*8 mm (premolars), and 6*10 mm (molars), with a total thickness of 80 microns. Positive and negative electrodes, crafted from copper wires, were independently routed and vertically integrated at the gold finger of the FPC. After connecting the FPC to the PCB, the entire aligner and circuit board were placed on the model. This assembly was then scanned using a 3Shape scanning device. The resulting model file was imported into EXOCAD to design the packaging mold, ensuring a mold gap of 0.3 mm was reserved. The designed mold was prepared by 3D printing, the final structure was packaged in PDMS with a protective layer measuring 0.3 mm in thickness.

### Piezoelectric Output Measurement

The piezoelectric performance was evaluated using a periodic impact component with a linear motor (NTI AG HS01−37 × 166) as the impact source. The output voltage signals of the components were measured using a low‐noise voltage preamplifier (Keithley‐6514 system electrometer from the United States). The output current signals of the components were measured using a low‐noise current preamplifier (Stanford Research SR570, USA). A data acquisition card (NI PCI‐6221) was utilized for signal collection and analysis. The piezoelectric outputs of PVDF‐TrFE/PVP composite fiber membranes, hollow pure PVDF‐TrFE fiber membranes, and composite fiber membranes with an addition of 0.5 wt.% Ti_3_C_2_TX MXene were measured. The fiber membranes were cut into squares of 4 * 4 cm, and after attaching copper foil electrodes, they were encapsulated using polyimide (PI) tape. The mechanical force applied was 1.5 N with a frequency of 15 Hz.

### Molecular Dynamics Simulation

The interactions between PVDF‐TrFE copolymer chains, PVP polymer chains, and Ti_3_C_2_TX MXene substrate were simulated using Materials Studio software. Each PVDF‐TrFE chain consists of 21 VDF and 9 TrFE monomers, with the monomers being randomly distributed within the PVDF‐TrFE chain (i.e., the molar concentrations of VDF and TrFE were 70 mol% and 30mol%, respectively). Similarly, each PVP chain contains 30 vinylpyrrolidone monomers. Initially, the interaction between Ti_3_C_2_TX MXene and 30 PVDF‐TRFE chains was calculated within a simulation box with periodic boundary conditions. The PVDF‐TrFE molecular chains were randomly placed above the Ti_3_C_2_TX MXene substrate within a 5 Å space. To study the restraining and compressing effects of PVP chains on PVDF‐TRFE chains, 30 PVP chains were randomly distributed above the PVDF‐TrFE chains for comparison. The simulation box was maintained at 298.15 K, with the number of atoms and volume also kept constant (NVT) throughout the calculation process. The timestep for the calculation process was 1 fs, simulating the molecular interactions and movements within the simulation box over 3 ns. Notably, during the simulation, the Ti_3_C_2_TX substrate was kept frozen, allowing the PVDF‐TrFE chains to move via van der Waals and electrostatic interactions with the nanoplate substrate.

### Finite Element Analysis

The effect of hollow voids and Ti_3_C_2_TX MXene on the deformation behavior of piezoelectric fibers under pressure was simulated using ABAQUS/Explicit software. To simplify the modeling complexity, models of pure PVDF‐TrFE fibers (PVDF‐TrFE), pure PVDF‐TrFE fibers with 0.5 µm hollow voids (h‐PVDF‐TrFE), and both types of fibers with added MXene (PVDF‐TrFE/MXene and h‐PVDF‐TrFE MXene) were established. The incorporation of MXene was simulated by increasing the Young's modulus of the fibers from 2000 to 2300 MPa. The deformation of the fibers was induced by applying a constant force to the upper and lower surfaces of the fibers via a plate.

### Materials' Characterization(Morphological Crystalline)

The surface and cross‐sectional morphology of the composite fibers were characterized using a field emission scanning electron microscope (FEI Instrument Co. Ltd.) and a transmission electron microscope (Thermo Scientific Talos F200i, USA). The diameter distribution of the hollow PVDF‐TrFE/MXene composite fibers obtained from the field emission scanning electron microscope images was statistically analyzed using Nano Measurer software. The crystal composition changes in the fibers after the introduction of hollow voids and Ti_3_C_2_TX MXene were studied using a Nicolet 6700 Fourier transform infrared spectrometer (Thermo Scientific Company, USA). Fourier transform infrared spectra were recorded from 4000 to 650 cm^−1^ in ATR mode, with a resolution of 4 cm^−1^, scanning 32 times. The crystal structure of the fibers after the introduction of hollow voids and Ti_3_C_2_TX MXene was investigated using an X‐ray diffractometer (Ultima IV, RIGAKU, Japan), with a scanning speed of 10°/min and a scanning step of 0.01°. Energy dispersive spectroscopy mapping (EDAX, USA) was used to characterize the distribution of C, Ti, and F atoms in the hollow PVDF‐TrFE/MXene composite fibers. X‐ray photoelectron spectroscopy (ESCALAB Xi+) were applied to study the functional group composition and the crystal structure of PVDF‐TrFE/MXene composite fiber.

### Tensile Testing

Tensile tests on the fiber membranes were conducted using an electronic universal testing machine (Instron, USA). The fiber membranes were cut into strips (1 cm × 5 cm) with an average thickness of 50 µm. Four types of test strips were prepared: PVDF‐TrFE/PVP composite fiber membranes, hollow pure PVDF‐TrFE fiber membranes, and composite fiber membranes with an addition of 0.5 wt.% Ti_3_C_2_TX MXene based on the previous two types. After measuring the dimensions, the fiber membranes were mounted on pneumatic grips with an exposed length of 2 cm. The tensile speed was set to 10 mm min^−1^, using a 500 N load sensor.

### Human Subject Study

A retrospective evaluation of the ARIA's effectiveness in occlusal status monitoring involved a dataset of 1467 patients who had sought orthodontic treatment at the West China Hospital of Stomatology over the period 2010–2022 (Institutional Review Board no. WCHSIRB‐D‐2021‐221). The inclusion criteria included: complete diagnostic information, no prior orthodontic treatment, no systemic syndromes, and possessing full permanent dentition (excluding third molars). Each participant underwent cephalometric examinations and had dental model records, with three experienced orthodontists independently diagnosing malocclusion. The dental models of these 1467 patients with a clear classification diagnosis of malocclusion were used to collect their occlusal data under ARIA.

For the identification of oral habits, 5 volunteers were recruited in the experiment testing device performance through a questionnaire among post‐graduate students from West China College of Stomatology. Among these, 3 participants were female and 2 were male, and the average age is 25.3±1.7 years old. All participating subjects of this research were informed, and written consent of all participants was obtained before the study. The ARIA system for the identification of oral habits was conducted in compliance with all the ethical regulations under a protocol that was approved by the Ethics Committee, West China School of Stomatology, Sichuan University, China.

### Machine Learning Algorithms for Classification and Prediction

The training was conducted using continuously measured occlusal information from 1467 different types of clinical tooth models on the articulator. The raw data obtained by the sensor undergoes meticulous data processing. The initial step is noise reduction, achieved through a low‐pass filter built into the ADC with a cutoff frequency of 20 Hz. Over time, the NCEN‐PENG experiences baseline drift due to charge accumulation. To correct this, we perform a Fourier transform to isolate signal components below 1 Hz and subsequently remove them, ensuring accurate baseline correction. The second step involves Independent Component Analysis (ICA). We utilize eight sensors to collect pressure data from tooth occlusion, recording mixed signals. To resolve this, we implement a fast ICA method to separate these signals and extract accurate pressure information for each tooth position, thereby significantly enhancing the prediction accuracy of the model. The third step is feature extraction, where it was derive specific features from each valid waveform. These features include the maximum and minimum values, peak‐to‐valley interval, number of zero crossings, number of inflection points, and the absolute square value. To minimize differences in applied force during articulator use across different groups, all features were normalized before entering the machine learning pipeline. This normalization ensured consistency for each subject during each test and enhanced the model's generalizability across the population. After data collection and analysis, the datasets for training and testing were shuffled and divided in an 80:20 ratio. Data points were randomly selected to ensure equal class representation. The ML model was developed to link occlusal features to electric signals and classify malocclusions based on medical records.

This paper refers to previous research^[^ ^61^ ^]^ and uses the PCA principle to combine the simulated signals of different types of malocclusion and the signals of bad oral habits x_i_ was merged into a set X:

(1) $$X\;=\left\{x_{1},x_{2},x_{3},\cdot \cdot \cdot ,x_{n}\right\}$$

Calculate the mean signal X_mean_:

(2) $$X_{mean}=\frac{1}{n}\;\sum_{i=1}^{n}x_{i}$$

Calculate the difference φi between the input signal and the mean signal:

(3) $$\varphi_{i}=X_{i}\;-X_{\mathrm{mean}}$$

The covariance matrix U can be calculated as:

(4) $$U=\frac{1}{n}\;\sum_{i=1}^{n}\varphi_{i}\varphi_{i}^{T}$$

The eigenvalues (λ_1_, λ_2_, λ_3_, …, λ_*k*_) (k is the rank of U) and eigenvectors (ω_1_, ω_2_,

ω_3_, …, ω_*k*_) can be obtained from the covariance matrix:

(5) $$U\;\omega_{i}=\lambda_{i}\;\omega_{i}\left(i\;=\;1,2,3,\cdot \cdot \cdot ,k\right)$$

Project all the original signal into a principal component matrix W

(6) $$y_{i}=W^{T}\;\left(x_{i}-X_{mean}\right)$$

By adjusting the k value, the rank of W is changed, and thus the dimension of the output is controlled. The principal component of the set X after dimensionality reduction is obtained from equation (6).

In this paper, t‐ ‐SNE was utilized to decrease the dimensionality of the data matrix derived from multi‐site sensor data to assess the correlation between different types of data x_i_ and x_j_.

Calculate the conditional probability that x_i_ and x_j_ are adjacent:

(7) $$p_{j|i}=\frac{\exp\left(-{\left\|x_{i}-x_{j}\right\|}^{2}/2\sigma_{i}^{2}\right)}{\sum_{k\neq i}\exp\left(-{\left\|x_{i}-x_{k}\right\|}^{2}/2\sigma_{i}^{2}\right)}$$

Symmetrized conditional probability(N is the total number of data):

(8) $$p_{ij}=\frac{p_{j/i}+p_{i|j}}{2N}$$

Using T distribution to calculate the similarity of low‐dimensional mapping:

(9) $$q_{ij}=\frac{{\left(1+{\left\|y_{i}-y_{j}\right\|}^{2}\right)}^{-1}}{\sum_{k\neq l}{\left(1+{\left\|y_{k}-y_{l}\right\|}^{2}\right)}^{-1}}$$

Adjust by gradient descent and other optimization algorithms to minimize the Kullback‐Leibler divergence C:

(10) $$C\;=\;KL\;\left(P∥Q\right)=\sum_{i\neq j}p_{ij}\;\log\frac{p_{ij}}{q_{ij}}$$

All training models were developed using Python (v.3.8), based on data collected from 1467 subjects experiencing with different malocclusion types, with a total of 60 000 s of ARIA recordings. The sensor signals were segmented using a sliding window with a sampling interval of 1 s for each malocclusion type representation. Various ML models were evaluated based on their precision–recall curves and F1‐scores, including XGBoost model, BP neural network, Decision Tree, MLP, KNN, SVM, and Random Forest.

### Development of the APP and Cloud‐Served Interface

The app was developed using Java on the Android Studio platform. The cloud‐based interface was designed by the Internet of Things software development team at Alibaba Cloud. The malocclusion classifications at the cloud server interface was categorized into An I, An II, An III, open bite and mandibular deviation based on the occlusal signals. The combined evaluation results of the 8 detection sites form five evaluation states. Those five malocclusion types were common in clinical evaluations, thus it could provide valuable insights into individual treatment planning.

## Conflict of Interest

The authors declare no conflict of interest.

## Supporting information



Supporting Information

Supplemental Movie 1

Supplemental Movie 2

Supplemental Movie 3

Supplemental Movie 4

## Data Availability

The data that support the findings of this study are available from the corresponding author upon reasonable request.
