# Supplementary material for: A Fully Integrated Orthodontic Aligner With Force Sensing Ability for Machine Learning‐Assisted Diagnosis
Source: Adv Sci (Weinh). 2024 Nov 19;12(2):2411187. doi: 10.1002/advs.202411187 (PMC11727240; doi:10.1002/advs.202411187)
Supplement: Supplementary file 1 — Supporting Information [file ADVS-12-2411187-s002.docx]

Supporting Information

A fully integrated orthodontic aligner with force sensing ability for machine learning-assisted diagnosis

Hao Feng^1^#, Wenhao Song^1^#, Ruyi Li^1^, Linxin Yang^1^, Xiaoxuan Chen^1^, Jiajun Guo^1^, Xuan Liao^2,3^, Lei Ni^2,3^, Zhou Zhu ^1^, Junyu Chen ^1^, Xibo Pei^1^, Yijun Li^1,4^*, Jian Wang^1^*

**Table of Content**

Note S1-S5 | Morphology, mechanical properties and crystalline characterization of fiber membranes

Table S1-S3 | Materials used, sensitivity, self-powered or not and self-powered voltage of the sensors in different operations

Figure. S1-S25

Supplementary References

Movie S1-S4

**Note S1 Morphology, mechanical properties and crystalline characterization of fiber membranes**

By adjusting the coaxial electrospinning process, piezoelectric fiber membranes with core-shell structures were obtained. Following the removal of PVP from the shell layer through ethanol ultrasonic etching, hollow-structured h-PVDF-TrFE/MXene (hPTM) piezoelectric fiber membranes were produced, as depicted in **Figure. S3a**. Statistical analysis revealed that the diameters of the core-shell structured fibers ranged from 0.3 to 1.1 micrometers, as shown in **Figure. S3b**. The energy dispersive spectroscopy (EDS) mapping in **Figure. S3c** displays the distribution of C, F, and Ti within the hollow fibers, indicating that the orderly arrangement of MXene and its uniform distribution in the shell matrix were well preserved during subsequent processing. The hollow structure of the fibers is clearly observable; for piezoelectric fibers with a diameter of 1 micrometer, the diameter of the hollow channels is approximately 0.2 micrometers.

The regular arrangement of MXene during the electrospinning process is a key factor in the ultra-high piezoelectric output of hPTM ENMs. To further elucidate the mechanism behind the orderly and uniform distribution of MXene within the shell matrix, we analyzed the h-PVDF/MXene fiber membranes using X-ray photoelectron spectroscopy (XPS). High-resolution XPS spectra of the C 1s region displayed characteristic peaks for C-C, C-F, C-Ti, and C-O bonds, located at 284.80, 290.16, 281.94, and 287.54 eV respectively (**Figure. S3d**). These results indicate that the functional groups on MXene's surface foster hydrogen bond formation, promoting the uniform distribution of MXene within the shell matrix.

The structural design and introduction of MXene fillers play a significant role in enhancing the formation of the β-phase in PVDF-TrFE, as determined by Fourier Transform Infrared Spectroscopy (FTIR) and X-Ray Diffraction (XRD) analyses before and after adding MXene, and with or without hollow structures. The FTIR results, shown in **Figure. S3e**, reveal vibration peaks attributed to the polar β-phase at 835 cm^-1^, 1268 cm^-1^, and 1410 cm^-1^. In samples without added MXene, less prominent peaks at 759 cm^-1^ and 975 cm^-1^ suggest the presence of the α-phase, which are absent in MXene-added samples. The XRD results (**Figure 3f**) further demonstrate MXene's capability to enhance the β-phase, with increased and intensified diffraction peaks at 2θ=20.41° corresponding to the β-phase (110) and (200) planes, even after etching PVP, the contributions to the PVDF-TrFE crystalline region transformation are well preserved, with no significant difference in the intensity of the β-phase diffraction peaks.

As shown in **Figure. S4a**, hollow piezoelectric fiber membranes, 50 μm thick and one-third the length of an Apple Pencil, can undergo deformations exceeding 1080 degrees, exhibiting exceptional softness and flexibility, suitable for oral movements such as chewing and grinding. Based on SEM (**Figure 4b**) and TEM (**Figure. S 4c**) images of magnified fiber membranes, we depicted a schematic of the distribution pattern of MXene within the shell matrix in **Figure. S4d**. The cross-sectional and two-dimensional planar images illustrate MXene's parallel alignment within the shell. During the spinning process, the inner PVP solution confines and compresses the shell components, while MXene's polarization locks the molecular chains, collectively promoting the formation of the β-phase.

Similarly, the introduction of hollow structures or MXene fillers significantly impacts the mechanical properties of the fiber membranes. When used as piezoelectric electronic devices, the fiber membranes must adapt to the forces and displacements generated by oral movements. We conducted tensile tests on four types of fiber membranes: core-shell PVP@PVDF-TrFE (PT), PT with introduced hollow structures (hPT), core-shell PVP@PVDF-TrFE/MXene (PTM), and PTM with introduced hollow structures (hPTM), as shown in **Figure. S4e**. The formation of hollow channels in the fiber membranes resulted in reduced strength and modulus, while the addition of MXene significantly increased the tensile strength and Young's modulus. This is attributed to the strong electrostatic forces between MXene and the shell matrix, and the regular, parallel arrangement of MXene serving as stress concentration points, dividing the fibers into multiple segments, thus greatly enhancing the fiber's mechanical properties. Therefore, despite the weakening caused by the formation of hollow channels, the MXene-enriched PVDF-TrFE fiber membranes, with their improved structure, exhibit considerable tensile strength and toughness, meeting the requirements for piezoelectric electronic devices in oral environments.

**Note S2 | Comparative Molecular Dynamics simulations of MXene, PVDF-TrFE and PVP**

To delve deeper into the interactions among components of the core-shell fiber and unveil the mechanisms behind MXene-induced local polarization and the confining effect of PVP, comparative Molecular Dynamics simulation (MDs) were performed using Ti_3_C_2_ MXene's periodic lattice and 20 PVDF-TrFE molecular chains (**Figure. S5a**). The results demonstrated that strong electrostatic interactions between MXene and PVDF-TrFE molecular chains drove the self-assembly of the piezoelectric fiber membrane. PVDF-TrFE molecular chains, constrained on the MXene surface, displayed polarization perpendicular to the MXene basal plane. Within 1 ns of the calculation's start, they were compressed within a space of approximately 30Å on the MXene surface, with this polarization-locking effect persisting throughout the simulation. PVP molecular chains were introduced to study the confining effect during spinning. Initially, 20 PVP chains were randomly distributed above the original system. Driven by van der Waals forces, the PVP aggregates moved downward, compressing the PVDF-TrFE molecular chains upon contact. In a 45° perspective view, the periodic lattice of MXene (green diamond shape) remained unchanged in size due to its fixed position in space, while the polymer molecular chains started to shrink as they moved closer to the MXene lattice. After 0.5 ns, this change was no longer significant, indicating that PVDF-TrFE was fully compressed and polarization-locked onto the MXene surface. To quantitatively study the confining effect on induced polarization, the adsorption energy of MXene to its surface-bound PVDF-TrFE molecular chains was calculated at 3 ns of the simulation (**Figure. S5b**), as well as the relative concentration of F along the direction perpendicular to the periodic lattice. Data on adsorption energy with and without the confining effect confirmed its contribution to the polarization of PVDF-TrFE. The translucent blue and red areas shown in **Figure. S5b** represent the distribution space of PVDF-TrFE at 3 ns, clearly showing PVP's compressive effect. The increase in adsorption energy was not significant, suggesting MXene's more substantial contribution to piezoelectric properties. **Figure. S5b** also indicated that the confining effect promoted out-of-plane polarization of PVDF-TrFE. Besides, the sharp rise of the curve at about 5Å indicated a highly oriented dipole perpendicular to the MXene basal plane, while subsequent fluctuations showed that this polarization effect extended along the perpendicular direction, indicating that microscale local induced polarization gradually expanded to macroscale polarization during spinning. MDs results further proved the dominant contribution of MXene to the piezoelectric properties of the hollow fiber membrane, with the PVP confining effect also playing an important role.

**Note S3 |** **Piezoresponse force microscopy (PFM)**

The piezoelectric and ferroelectric properties of hollow fiber membranes were investigated using Piezoresponse force microscopy. Amplitude (**Figure. S6a**) and phase (**Figure. S6b**) images revealed the magnitude and orientation of polarization, with brighter areas indicating higher piezoelectric responses^1^. This suggests the presence of local polarization induced by MXene, as β-phase crystals are locked on the surface of MXene^2^, leading to more pronounced electrostriction in PVDF-TrFE. The displacement under an electric field decreases further away from the MXene, indicating that the local polarization induced by MXene gradually extends to a macroscopic net polarization^3^. The PFM amplitude and phase images underscore the significant role of MXene in enhancing the piezoelectric response of the fibers.

The polarization of the sample can be switched by an electric bias^4^, displaying characteristic phase hysteresis loops and butterfly-shaped amplitude loops, demonstrating the piezoelectric and ferroelectric characteristics of the fibers. The butterfly-shaped amplitude loop in **Figure. S6c** reveals the piezoelectric nature of the fibers, with the two transition points at the bottom indicating the switching behavior of dipoles^5^. When a sufficiently strong electric field is applied, polarization is switched, showing a low coercive field. This can be attributed to the orderly distribution of MXene during the spinning process, without aggregation, as evident from the significant increase in Young's modulus after the addition of MXene. The favorable orientation of β-phase crystals facilitates the switching of dipoles^6^. Moreover, MXene uniformly distributed in PVDF-TrFE enhances the fiber's AC conductivity under an electric field, optimizing polarization efficiency. The hysteresis loop in **Figure. S6d** shows the saturation of PFM phase at higher voltages in the fibers and a 180° phase switch under reversed electric bias, confirming the ferroelectricity of the fibers^7^. The presence of an internal field causes the difference in positive and negative coercive voltages, with the direction of this internal field corresponding to the alignment of the dipoles. The strong local electrostatic field formed by MXene leads to the self-polarization of the fluoropolymer, and the internal field present in the self-polarized crystals is responsible for the shift in the phase loop^8^. The piezoelectric coefficient d_33_ is determined using static-sensitivity-based quantification method through the following equation^9^:

$$d_{33}=\frac{A_{HSPR}S_{D}}{F_{p}V_{HSPR}}$$

where *A_HSPR_* is the amplitude (mV), *S_D_* is deflection sensitivity (nm V^−1^),Fp is the facility parameter, and *V_HSPR_* represents the external applied AC voltage (V). The tip was calibrated by measuring the standard polarized PVDF sample with a known piezoelectric constant and the *S_D_/ F_p_* value of the tip was determined to be 0.0166. The piezoelectric coefficient of hollow fiber membrane could be accordingly obtained as around – 58.6 pm V^−1^

**Note S4** **| Comparison of the sensors**

Piezoelectric, triboelectric, capacitive, and piezoresistive sensors are common types of flexible strain sensors. Capacitive and resistive sensors require an additional voltage in the circuit. When subjected to external forces, the current signal in the circuit changes due to variations in the sensor's capacitance and resistance. This change is measured and analyzed to understand the characteristics of the external force. Consequently, these sensors necessitate additional circuit design and a power supply for their operation.

In contrast, piezoelectric and triboelectric sensors offer the advantage of being self-powered. They can generate electrical signals under external forces without the need for an additional voltage. This self-powered feature means low power consumption and lightweight, making them naturally advantageous for wearable electronic devices. Wearable electronics that do not require external power supply are crucial for promoting a low-carbon lifestyle.

In **Table S1**, we compare the four types of sensors across various studies. For the self-powered sensors, their output voltage data are also listed. Our piezoelectric sensor exhibits self-powered characteristics and offers higher output voltage and sensitivity compared to other self-powered sensors.

Lu et al^10^, measured and compared the sensitivity of their triboelectric sensor with the other three types of sensors. Similar to the triboelectric sensor, our piezoelectric sensor demonstrates higher sensitivity at a lower cost (**Table S2**). However, the self-powered voltage of the triboelectric sensor, as shown in **Table S1**, is lower than that of our piezoelectric sensor.

**Note S5** **| Malocclusion Classification experiment based on ML**

Backpropagation (BP) neural network is a fundamental type of artificial neural network that uses the backpropagation algorithm for training. It consists of an input layer, one or more hidden layers, and an output layer. In training, the network processes data in a forward pass to make predictions, calculates errors using a loss function, and then adjusts its weights during a backward pass using calculated gradients. This iterative adjustment helps minimize prediction errors, enhancing the model's accuracy. BP neural networks are widely used in applications such as image recognition, natural language processing, and predictive analytics due to their effectiveness and adaptability. In the training of malocclusion classification using ARIA, the accuracy of model predictions increases with the number of training iterations, usually reaching a peak at 100 iterations. The curvature of the accuracy curve converges gradually, as verified by the normalized loss over 100 iterations(**Figure. S12a,b,d,e,g,h**). The model achieves an accuracy of 0.645 based on maxillary data, 0.735 based on mandibular data, and 0.934 when both maxillary and mandibular features are included. The confusion matrix presents the prediction accuracy for different classifications using the BP neural network(**Figure. S12c,f,i**). The findings demonstrate that as the number of included features increases, the model's accuracy significantly improves.

XGBoost is a parallel tree-boosting method designed to solve large-scale problems with enhanced accuracy. It is a classifier that originates from gradient boosting, where weak tree classifiers are combined iteratively to create a robust classifier. Residual errors are computed for each tree to reduce the error margin for the following tree, resulting in a decreased overall error rate. Each tree plays a part in developing a strong boosted classifier through the formation of an ensemble of weak classifiers. The confusion matrix is a pivotal metric in evaluating the concordance between empirical data and the predictions generated from ML algorithms, succinctly summarizing the model’s predictive accuracies. This matrix underpins the assessment of accuracy across detection and cross-validation phases. The nuanced evaluation of model efficacy leverages receiver-operator characteristic (ROC) curves and AUC indices, where the ROC curve provides an illustrative summary of a binary classifier's diagnostic abilities, delineating sensitivity (true positive rate) and specificity (1–false positive rate). The classification results of the five types of malocclusion determined by applying XGBoost are presented in a confusion matrix, which confirmed that malocclusions in the same group were also classified. The evaluation of performance metrics demonstrates a distinct increase in accuracy depending on the data used: 0.795 accuracy is achieved with the inclusion of maxillary data alone, (**Figure. S13a**) 0.812 with only mandibular data, (**Figure. S13c**) and a notable 0.955 when both data sets are combined. (Figure. 5c) This tiered accuracy underscores the significant impact of integrating comprehensive dental data in classification processes. The illustration presents the ROC curves of the XGBoost algorithm evaluated on three distinct data scales, (Figure.5b, **Figure. S13b,d**) complemented by the corresponding AUC results. After integration of 16 channels from maxillary and mandibular data, the AUC values for XGBoost consistently exceed 0.9, indicating robust model performance.

The Multilayer Perceptron (MLP) is a type of feedforward artificial neural network designed to transform input vectors into output vectors. Structurally, the MLP is analogous to a directed graph, composed of multiple layers of nodes, with each layer fully connected to the following one. Beyond the input layer, each neuron is equipped with a nonlinear activation function and is predominantly trained using the backpropagation technique, enhancing its capability to handle complex patterns. The confusion matrix indicates that MLP performs worse than SVM, XGBoost, and RF in malocclusion classification. Specifically, the accuracy with only maxillary data is 0.743,(**Figure. S14a**) with only mandibular data it drops to 0.600,( **Figure. S14b**) and with both data sets combined, it improves to 0.892. (**Figure. S14c**) This pattern aligns with those observed in other machine learning models. The ROC curves are indicated in **Figure. S14d-f**. The AUC value for MLP exceeds 0.9 for maxillary data, but falls to 0.894 for mandibular data, improving significantly to 0.983 when both data types are combined.

The K-Nearest Neighbors (KNN) algorithm is a foundational tool in both classification and regression scenarios. It is implemented through the KNeighborsClassifier, which allows for customization of several parameters including the K value, distance metric, weighting scheme, and computational algorithm. The K value stands out as particularly pivotal, as an overly large K value can reduce model complexity and lead to underfitting, while an exceedingly small K value can heighten sensitivity and risk overfitting. To address this, a meticulous optimization process involving the grid search strategy and cross-validation from the Scikit-learn library is utilized to precisely identify the best K value. As indicated by the confusion matrix, KNN ranks below XGBoost but above MLP in terms of malocclusion classification effectiveness. Specifically, it achieves an accuracy of 0.739 with maxillary data (**Figure. S15a**) and 0.708 with mandibular data,( **Figure. S15b**) which significantly improves to 0.902 when both datasets are utilized—Figures closely aligning with those of MLP.( **Figure. S15c**) ROC curved were indicated in **Figure. S15d-f**. Meanwhile, MLP's AUC is 0.91 for maxillary data and dips to 0.898 for mandibular data, yet it escalates impressively to 0.974 with the integration of both datasets.

The decision tree (DT) is a versatile machine learning technique, renowned for its robust data searching and modeling capabilities. Employing both classification and regression as nonparametric models, DTs offer a distinct advantage due to their transparency, which simplifies the understanding and interpretation of the analytical process for researchers. Furthermore, decision trees can be developed quickly, making them ideal for enabling timely, short-term predictions. According to the confusion matrix, DT is the least effective of the six machine learning models in classifying malocclusions. It registered an accuracy of 0.694 with maxillary data (**Figure. S16a**) and 0.739 with mandibular data. (**Figure. S16b**) When both data types are utilized, accuracy climbs to 0.822, (**Figure. S16c**)which remains inferior to XGBoost's results using single jaw data alone. ROC curved were indicated in **Figure. S16d-f.** Additionally, MLP's AUC for maxillary data was 0.81 and fell to 0.84 for mandibular data; despite combining these datasets, the AUC did not surpass 0.9.

Support Vector Machines (SVM) epitomize an advanced supervised learning technique designed to enhance algorithmic generalization through the reduction of structural risks. This methodology is extensively applied in the realms of statistical classification and regression analysis. Within Scikit-learn, the SVM algorithm library is methodically organized into two primary segments: classification and regression. The classification segment houses the SVC, NuSVC, and LinearSVC algorithms, whereas the regression segment includes the SVR, NuSVR, and LinearSVR algorithms. This paper specifically employs the SVC classification algorithm to address the classification challenges posed, given the ambiguous linear separability of the dataset. The SVM's performance in malocclusion classification was inferior to that of XGBoost, the accuracy rates are 0.805 with maxillary data only,(**Figure. S17a**) 0.781 with mandibular data, (**Figure. S17b**) and an impressive 0.948 (**Figure. Sc**) when both data sets are utilized, values that closely match those of XGBoost. ROC curves of the SVM algorithm evaluated on three distinct data scales are presented in **Figure. S17d-f .** The AUC value of SVM is similar to that of XGBoost. Under the three data models, it always exceeds 0.9, indicating that the model performance is robust. Incorporating both maxillary and mandibular data into the analysis allows the AUC value to exceed 0.98.

The random forest(RF) algorithm stands out as an exceptionally flexible machine learning methodology, endowed with vast potential for diverse applications. The training process for the random forest model distinctively involves the use of the random forest classifier function from the Scikit-learn library. Owing to its tolerance towards variations in hyperparameter settings, this algorithm not only facilitates but also streamlines the model development process. The confusion matrix demonstrates that RF outperforms SVM but does not reach the high standards set by XGBoost in classifying malocclusions. It achieves accuracy rates of 0.778 with maxillary data only,( **Figure. S18a**) 0.819 with mandibular data,( **Figure. S18b**) and an outstanding 0.955 with both datasets(**Figure. S18c**)—results that are closely aligned with those of XGBoost. The ROC curves were indicated in **Figure. S18d-f**. RF's AUC values, mirroring those of XGBoost, consistently surpass 0.9 across the three data models, affirming its robustness. Furthermore, the amalgamation of maxillary and mandibular data escalates the AUC to an impressive 0.996.

**Table S1|** Materials used, sensitivity, self-powered or not and self-powered voltage of the sensors in different operations

| **Ref.** | **Materials** | **Sensitivity** | **Self-power** | **Mechanism** |
| --- | --- | --- | --- | --- |
| This work | PVDF-TrFE, Ti_3_C_2_ MXene | 0.366 V/N/ 0.5856 V/kPa | Yes(78.5 V) | Piezoelectric |
| 1^11^ | PVDF | 1.025–9.875 V/N | Yes(8.45 V) | Piezoelectric |
| 2^12^ | PVDF, Ti_3_C_2_ MXene | 0.01845 V/kPa | Yes(3.15 V) | Piezoelectric |
| 3^10^ | PVC, nylon | 0.376 V/N | Yes(3.08 V) | Triboelectric |
| 4^13^ | Gelatin, agar hydrogel, seaweed | 0.28  V/kPa | Yes(3 V) | Triboelectric |
| 5^14^ | Nylon, copper, PTFE | 0.048  V/kPa | Yes (3 V) | Triboelectric |
| 6^15^ | Carbon nanotubes, silica gel | 0.35  V/N | Yes(4.4  V) | Triboelectric |
| 7^16^ | Disulfide CANs | 9.26 kPa^-1^ | No | Capacitive |
| 8^17^ | Ecoflex | 0.292 kPa^-1^ | No | Capacitive |
| 9^18^ | PI | 0.077 kPa^-1^ | No | Capacitive |
| 10^19^ | CNFs/MWCNTs | 4.11 kPa^-1^ | No | Piezoresistive |
| 11^20^ | P(St-MAA) | 13.65 kPa^-1^ | No | Piezoresistive |

**Table S2|** Sensitivity, trigger point, cost of different sensors

| **Ref.** | **Sensitivity** | **Trigger point** | **Cost** | **Mechanism** |
| --- | --- | --- | --- | --- |
| ^10^ | 0.12112 kPa-1 | 1 N | ￥288/ ＄39.7 | Capacitive |
|  | 0.0107 kPa-1 | 4 N | ￥13/ ＄1.8 | Piezoresistive |
|  | 0.00212 V/kPa | 10 N | ￥12/ ＄1.7 | Piezoelectric |
|  | 0.61183 V/kPa | 1 N | ￥2/ ＄0.3 | Triboelectric |
| This work | 0.5856 V/kPa | 1 N | ￥2/ ＄0.3 | Piezoelectric |

**Table S3 | List of ML accuracy for flexible mechanical sensing using wearables.**

| **Ref.** | **Sensors** | **ML algorithms** | **Subjects** | **Classify Accuracy** | **Applications** |
| --- | --- | --- | --- | --- | --- |
| ^21^ | A triboelectric sensor | ANN | NA | NA | Blood pressure estimation |
| ^22^ | A triboelectric sensor | DT | NA | 98% | Speaker/voice commands recognition |
| ^23^ | Piezoresistive sensors | CNN | NA | 70% | Silent speech recognition |
| ^24^ | Piezoresistive sensors | RF and ANN | NA | 97% | Human posture/motion identification |
| ^25^ | Triboelectric sensors | CNN | 10 | 96% | Human posture/motion  Identification |
| ^26^ | Triboelectric and piezoelectric sensors | LDA | NA | 93% | Sign language recognition of 26 letters |
| ^27^ | Multi-channel piezoelectric sensors | GMM+EM | NA | 97.5% | Speaker recognition |
| ^28^ | Mechanoacoustic sensors | CNN+KNN | 16 | 91% | Speaker recognition |
| This work | 16-channal piezoelectric sensor | XGboost | 1467 | 95% | Malocclusion classification |

ANN, artificial neural network. DT, decision tree. CNN, convolutional neural network. RF, random forest. LDA, linear discriminant analysis. GMM, gaussian mixture model. EM, expectation maximization. KNN, k-nearest neighbor. XGboost, extreme gradient boosting


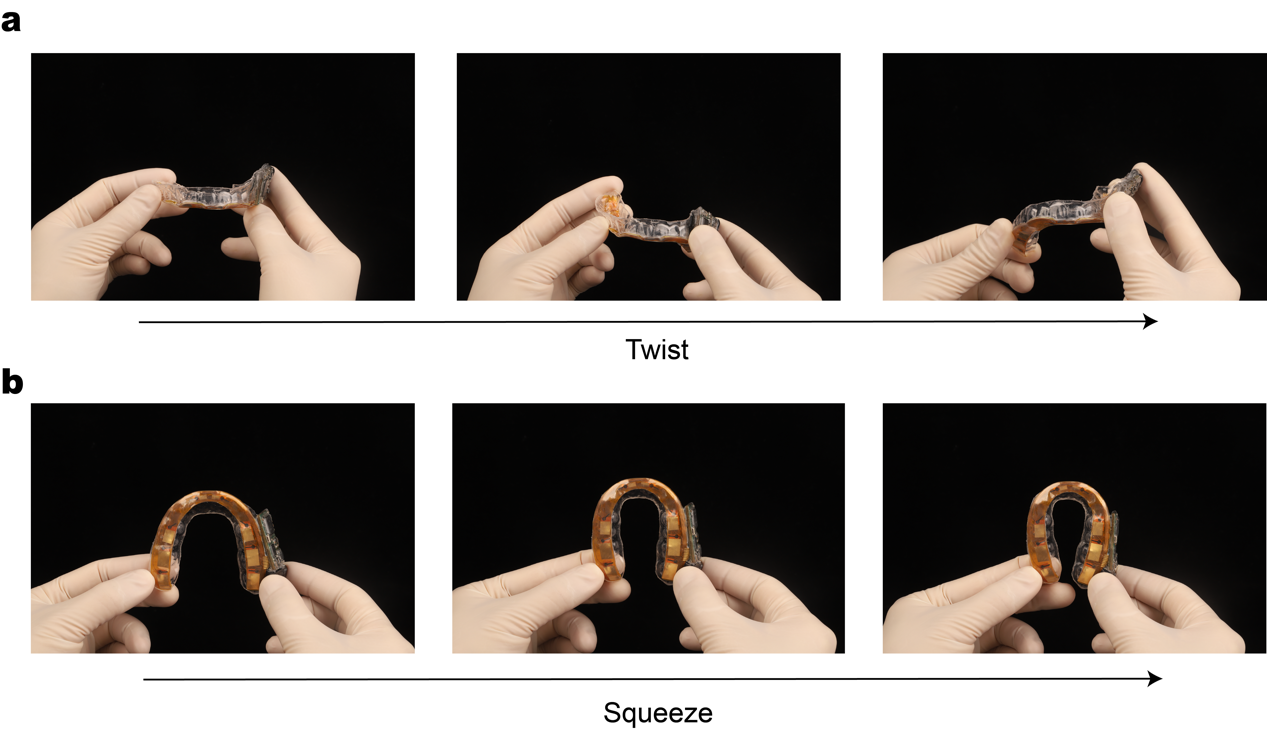


**Figure. S1|** **Characterization of ARIA flexibility a**, Photographs of ARIR twisting up and down. **b**, Photographs of ARIR in the process of squeezing towards the middle.


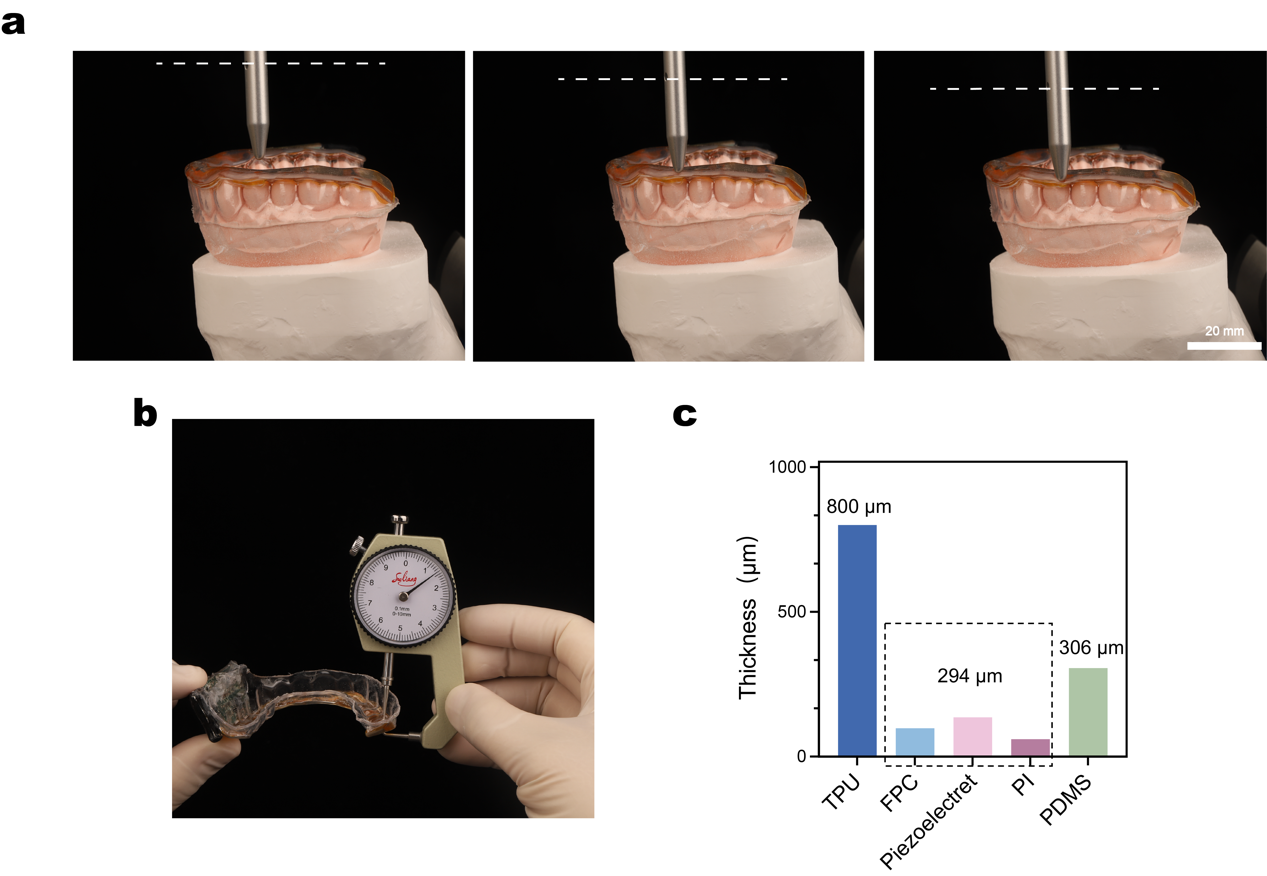


**Figure. S2|** **Elasticity and thickness of ARIA a**, optical image of ARIA occlusal surface deformed after pressure. **b**, Photograph of measuring the thickness of the ARIA occlusal surface using a telemeter. **c**, Average thickness of each layer of ARIA


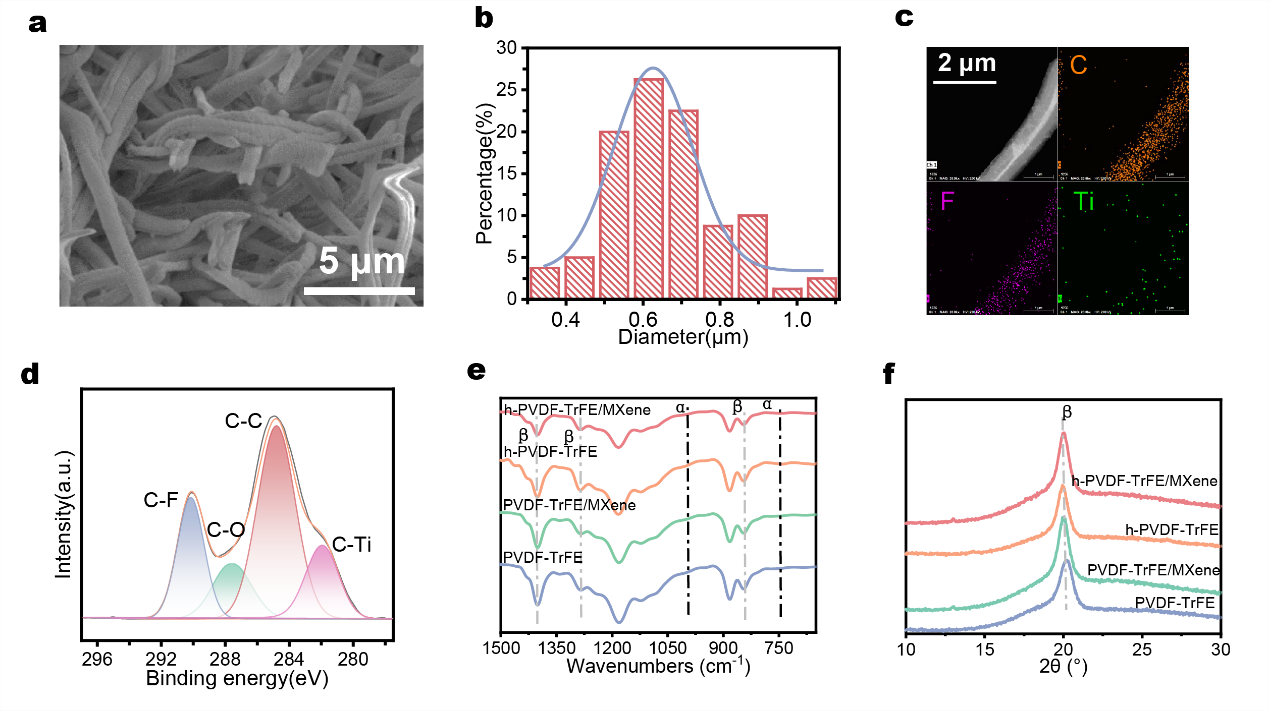


**Figure. S3|** **Morphology and crystalline characterization of fiber membranes** **a,** SEM, **b,** Diameter distribution, and **c,** EDS-mapping of h-PVDF-TrFE/Mxene nanofibers, EDS-mapping of the hollow fiber showing the homogeneous distribution of C F and Ti in the PVDF matrix. **d,** XPS spectra of C 1s revealed characteristic peaks of C-C, C-F, C-Ti, and C-O bonds, located at 284.80, 290.16, 281.94, and 287.54 eV respectively. Interactions between functional groups on MXene's surface and PVDF-TrFE promote hydrogen bond formation, ensuring uniform distribution of MXene in the shell matrix. **e,** FTIR. Vibration peaks attributable to the polar β-phase at 835 cm^-1^, 1268 cm^-1^, and 1410 cm^-1^. In samples without MXene, less pronounced peaks at 759 cm^-1^ and 975 cm^-1^ indicated the presence of the α-phase, which was absent in MXene-added samples. **f,** XRD. MXene can induce the β-phase, with increased intensity of the diffraction peak at 2θ=20.41° for the β-phase (110) and (200) lattice planes in MXene-added samples.


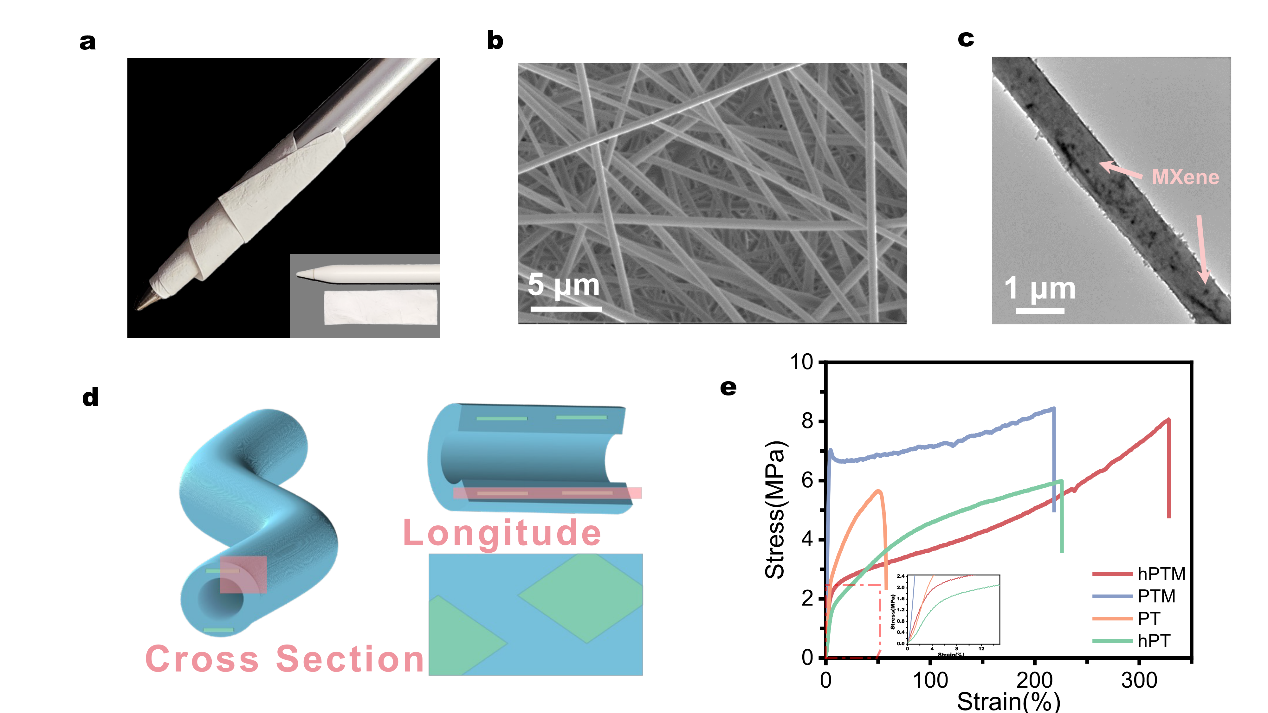


**Figure. S4|** **Demonstration, mechanical properties and fillers distribution of** **fiber membranes a,** Flexibility and softness of hPTM fiber membrane. **b,** SEM image of the core-shell fibers (scale bar is 5 μm). **c,** TEM image of the electrospun PTM nanofibers showing parallel distribution of MXene nanosheets in PVDF-TrFE matrix (scale bar is 1 μm). **d,** Theoretical schematic of parallel arrangement of MXene in hollow fibers. **e,** Stress-strain curve of fiber membranes.


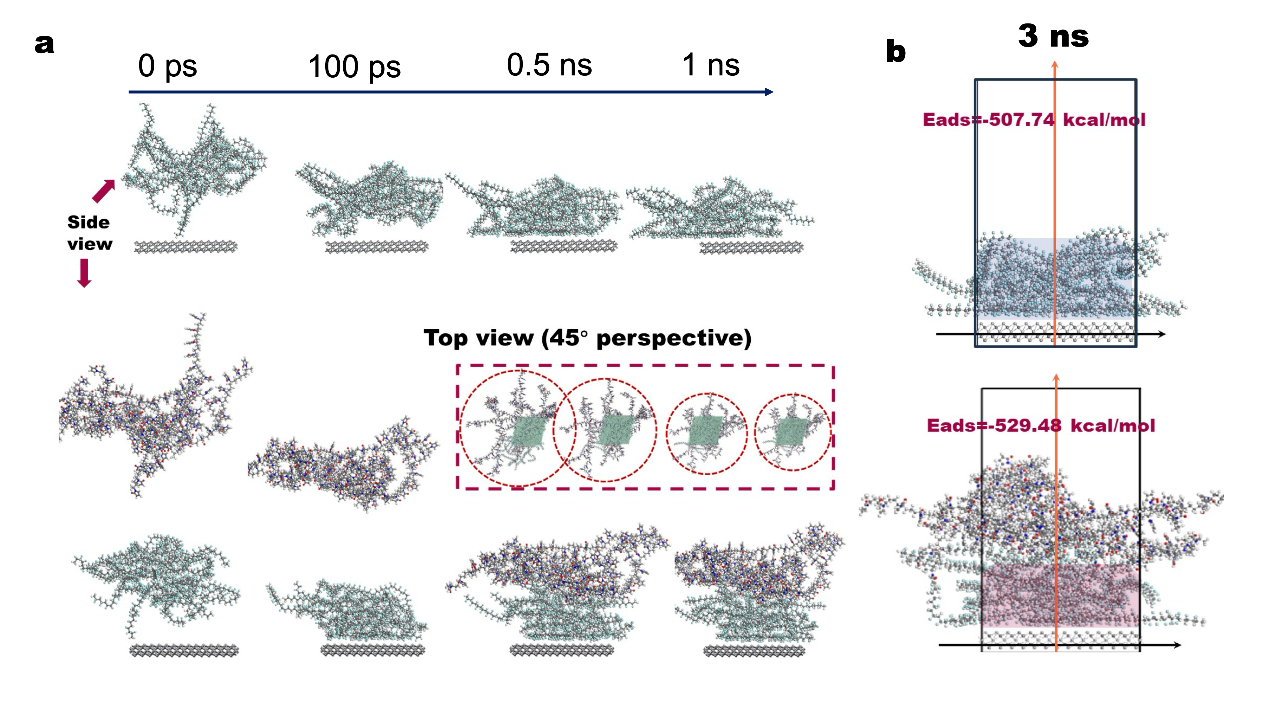


**Figure. S5|** **Comparative molecular dynamics simulations a.** Comparative MD simulations of the polarization of PVDF-TrFE copolymers on the Ti_3_C_2_ substrate, which is enhanced by confinement effect effects, as seen in **b.** the adsorption energy calculated from snapshots of molecular motion at 3 ns


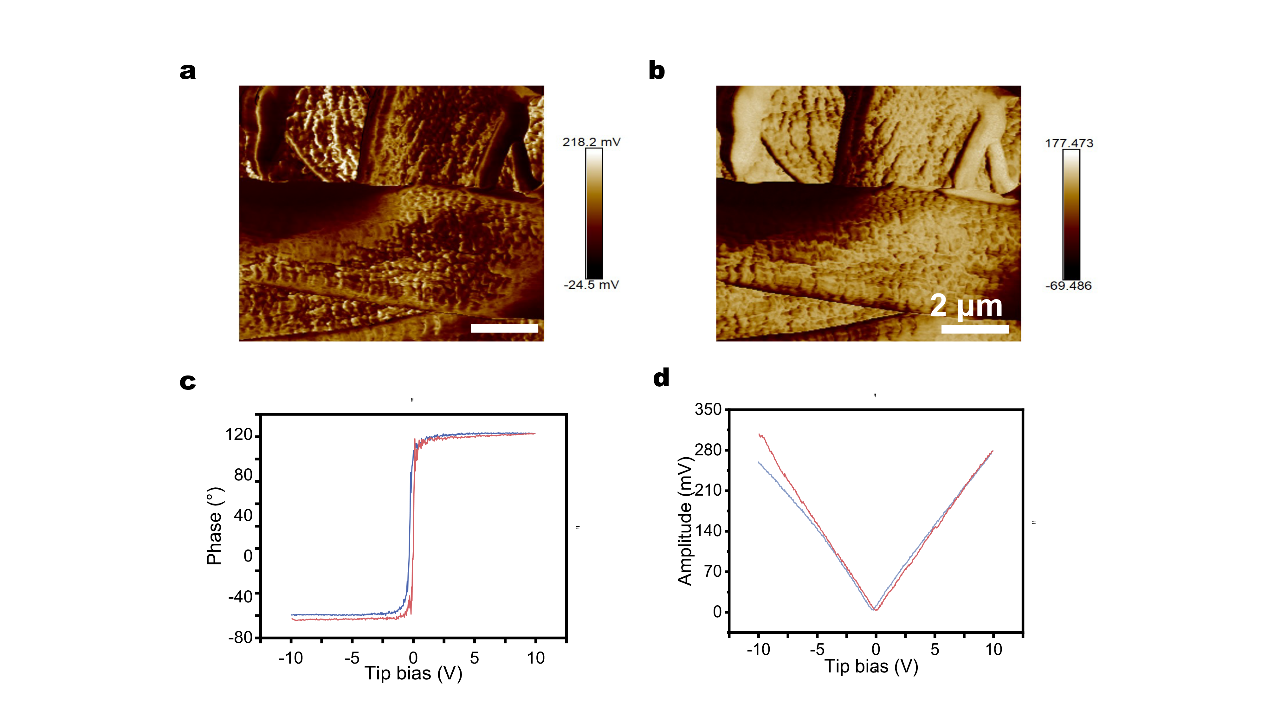


**Figure. S6| Piezoresponse force microscopy of** **the hPTM ENMs** **a,** The piezoresponse force microscopy (PFM) amplitude and **b,** phase difference images **c,** The amplitude of the ferroelectric hysteresis loop as a function of the applied voltage and **d,** a rhombus-shaped curve with a phase difference of 180° as a function of the applied voltage.


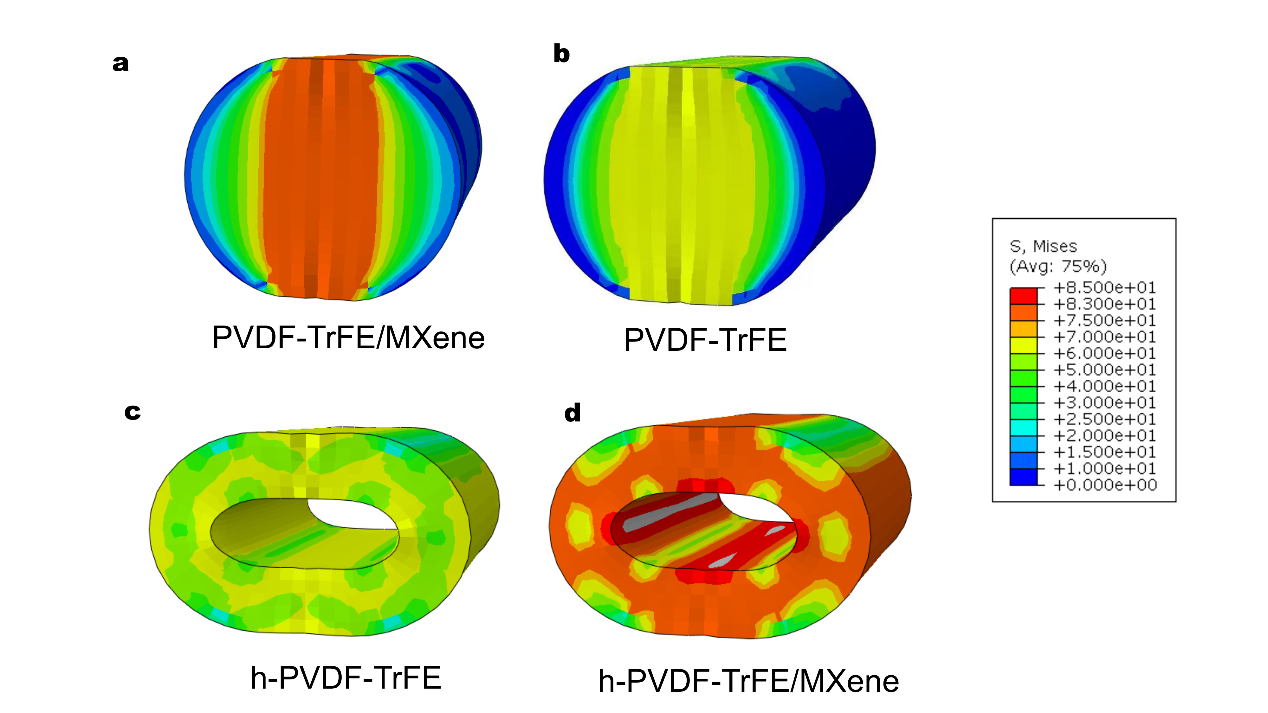


**Figure. S7|** **FE analysis of fibers** Stresses and deformations of fiber parts when a flat plate is pressed on the top and bottom surfaces of the fiber models of **a,** PTM **b,** PT **c,** hPT **d,** hPTM


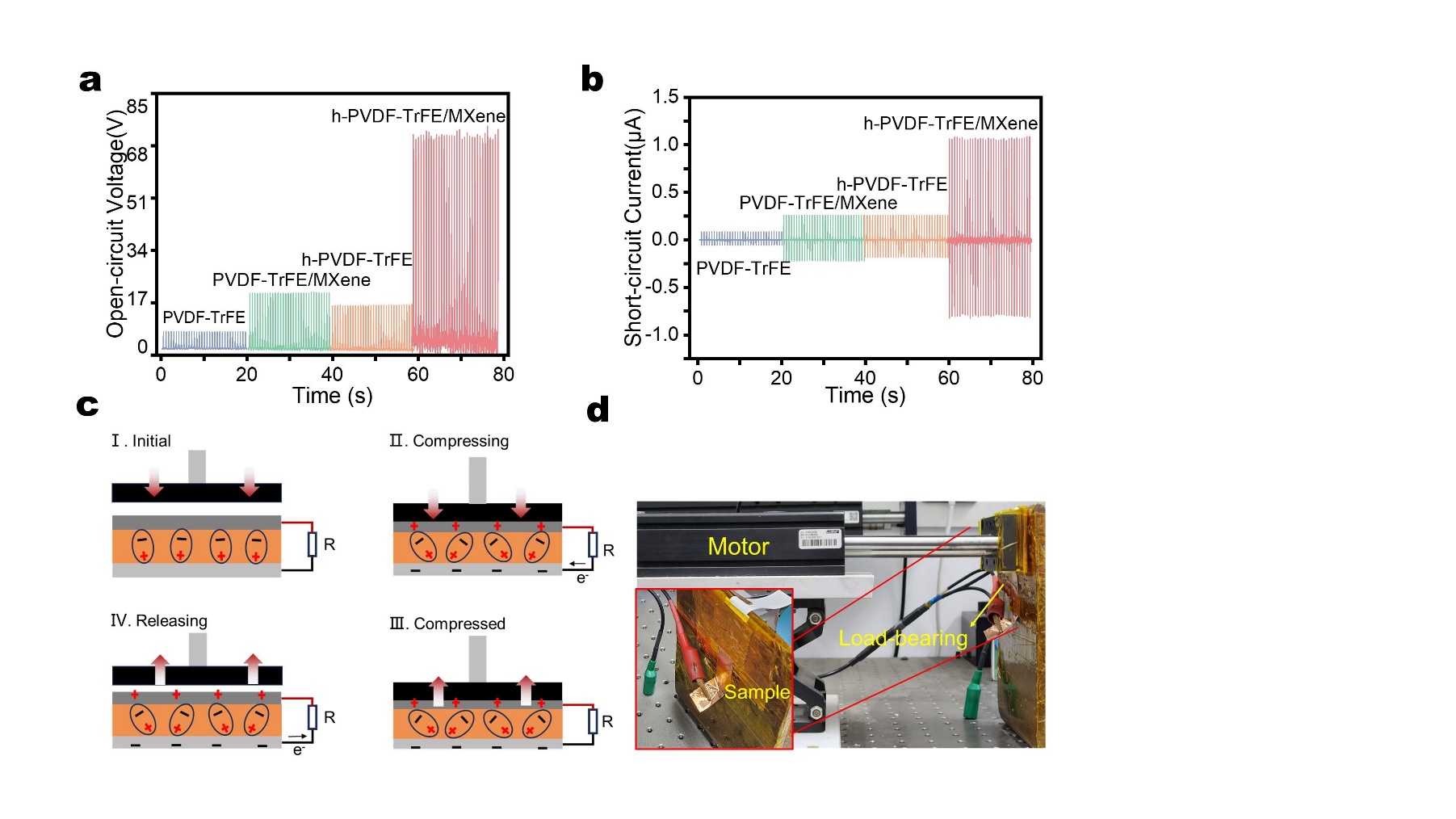


**Figure. S8|** **The principles and procedures of piezoelectric output measurement** **a,** Multiperiod open-circuit voltages and **b,** short-circuit currents of fiber membranes. **c,** Schematic diagram of piezoelectric signal generation. **d,** Schematic diagram of the piezoelectric output measurement setup.


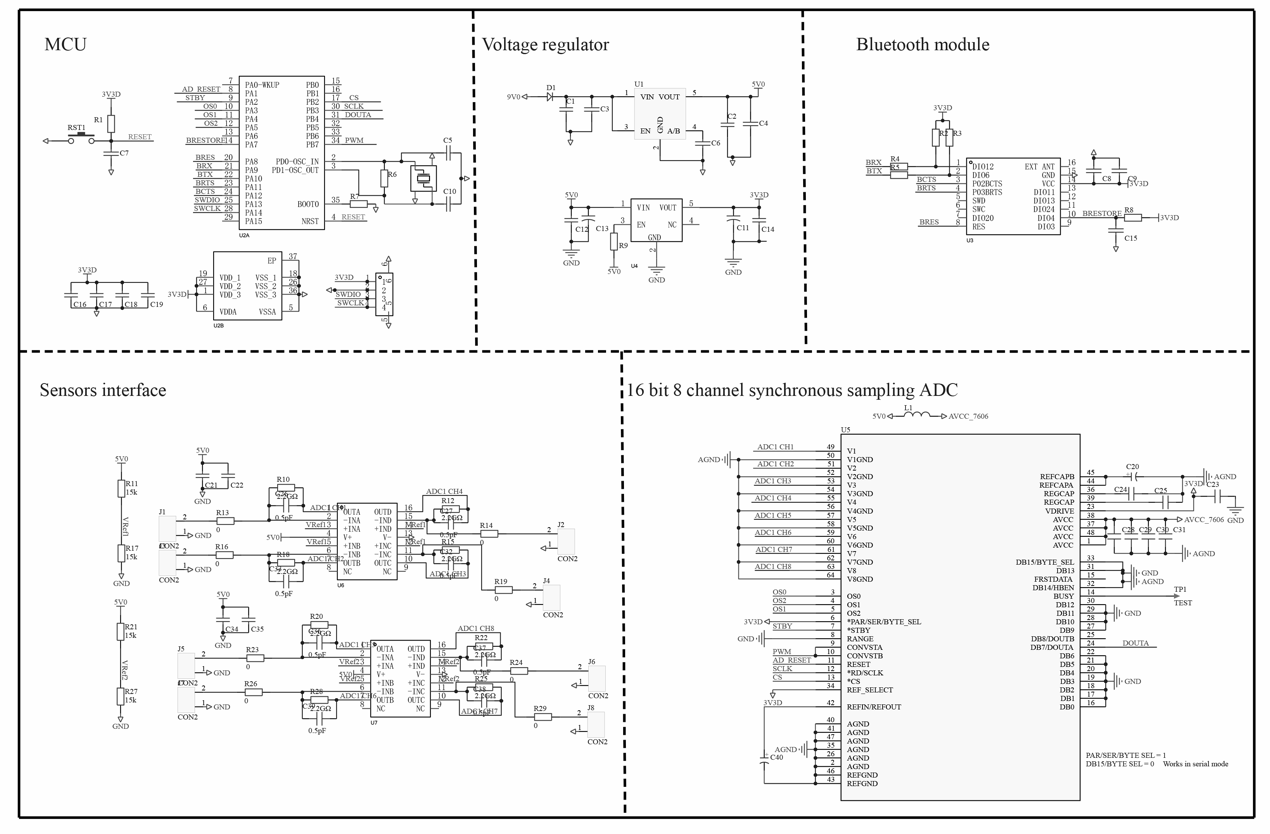


**Figure. S9| Circuit design for the ARIA.**

**
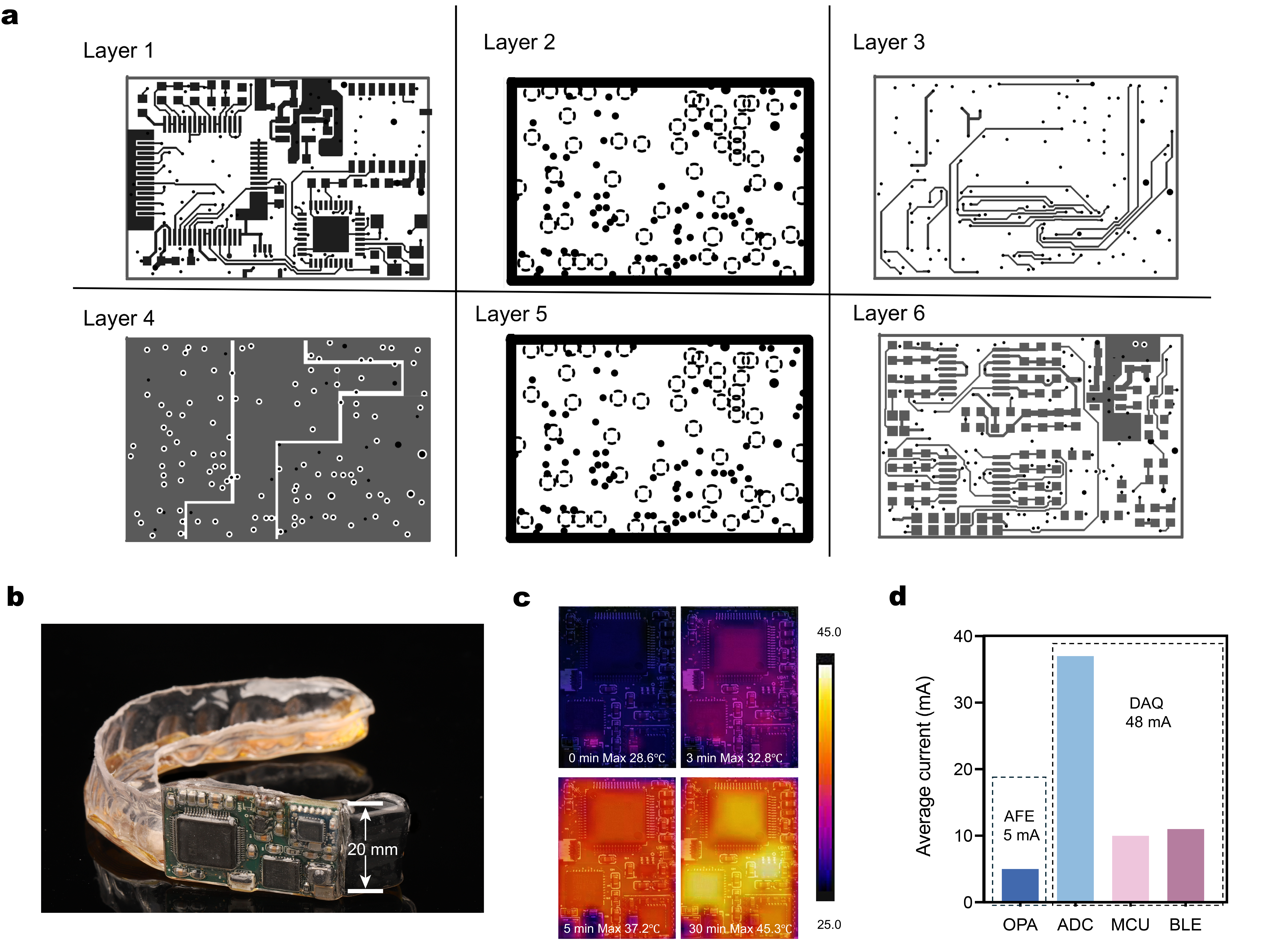
**

**Figure. S10|** **Circuit schematic and characteristics**. **a**, The 6-layer structure of printed circuit board. **b**, The photograph of ARIA. **c**, Thermal photos of the circuit board after varying periods of operation. **d**, current consumption of the circuit components with a 6 V input. The total average current consumption is 53 mA.

**
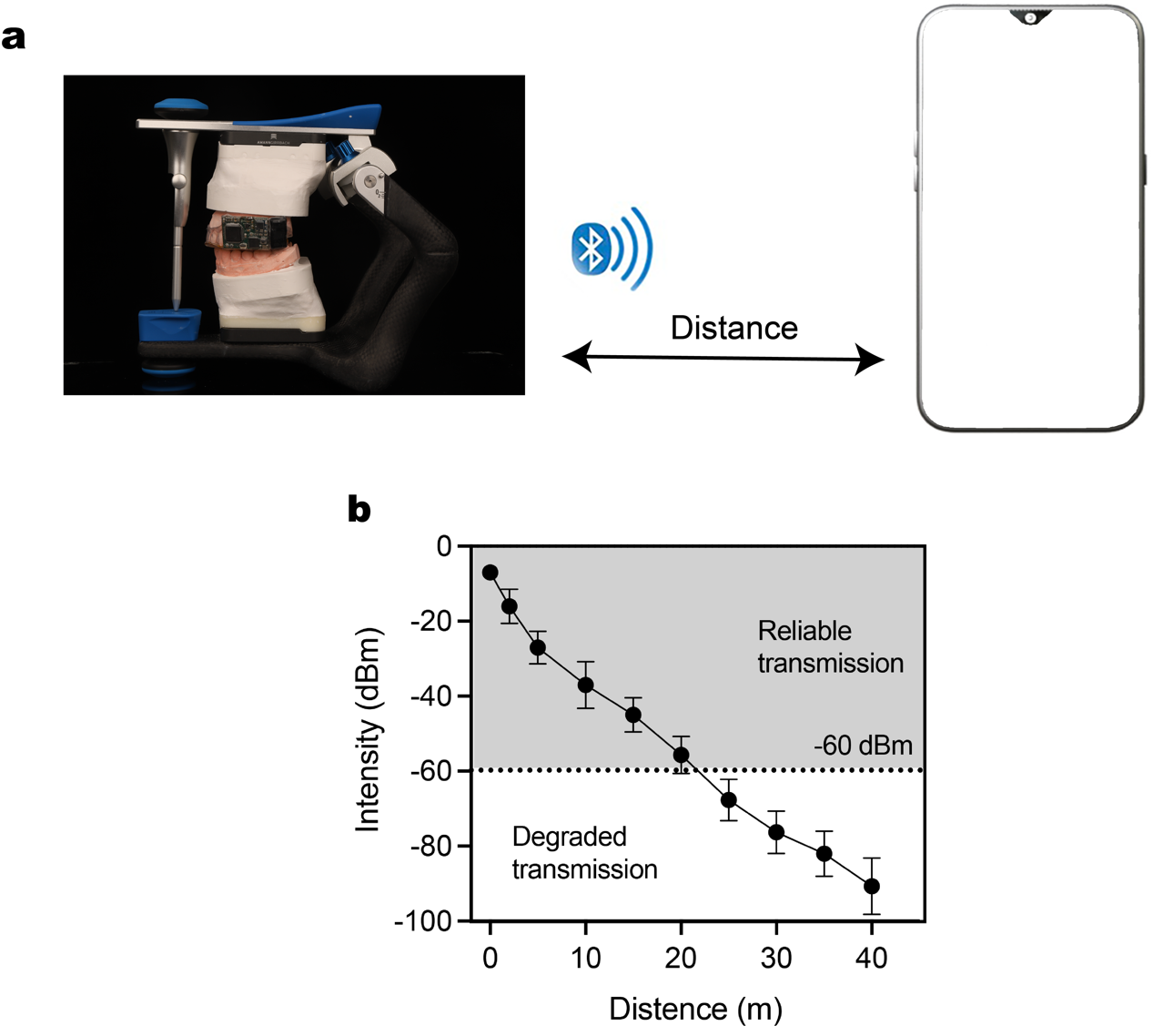
**

**Figure. S11| Wirelessly transmission ARIA's piezoelectric signals via Bluetooth. a,** The testing setup showing data transmission between the ARIA and a smartphone. **b**, Bluetooth signal intensity with increasing transmission distance.

**
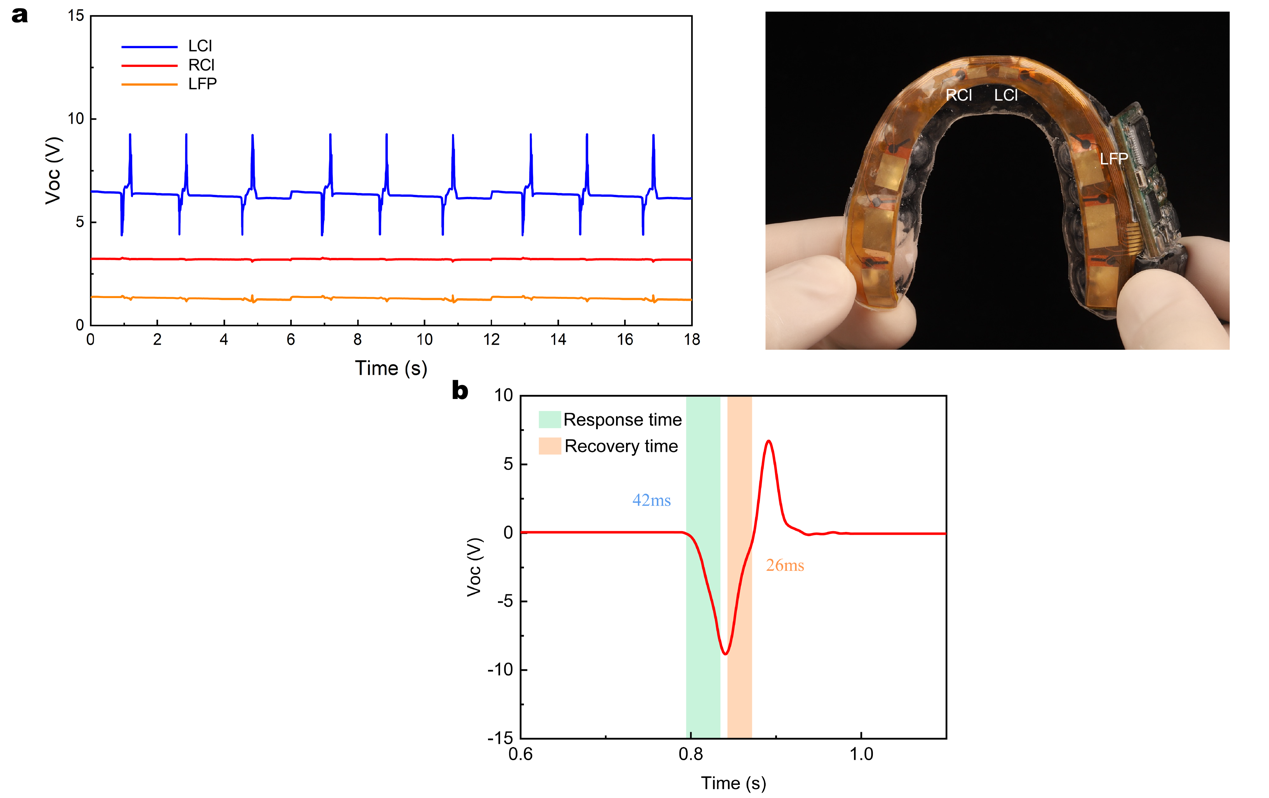
**

**Figure. S12|** **Performance of the monitoring system. a,** Voltage response obtained from LCI, RCI, and LFP when pressing LCI. **b**, Response time and hysteresis time.

**
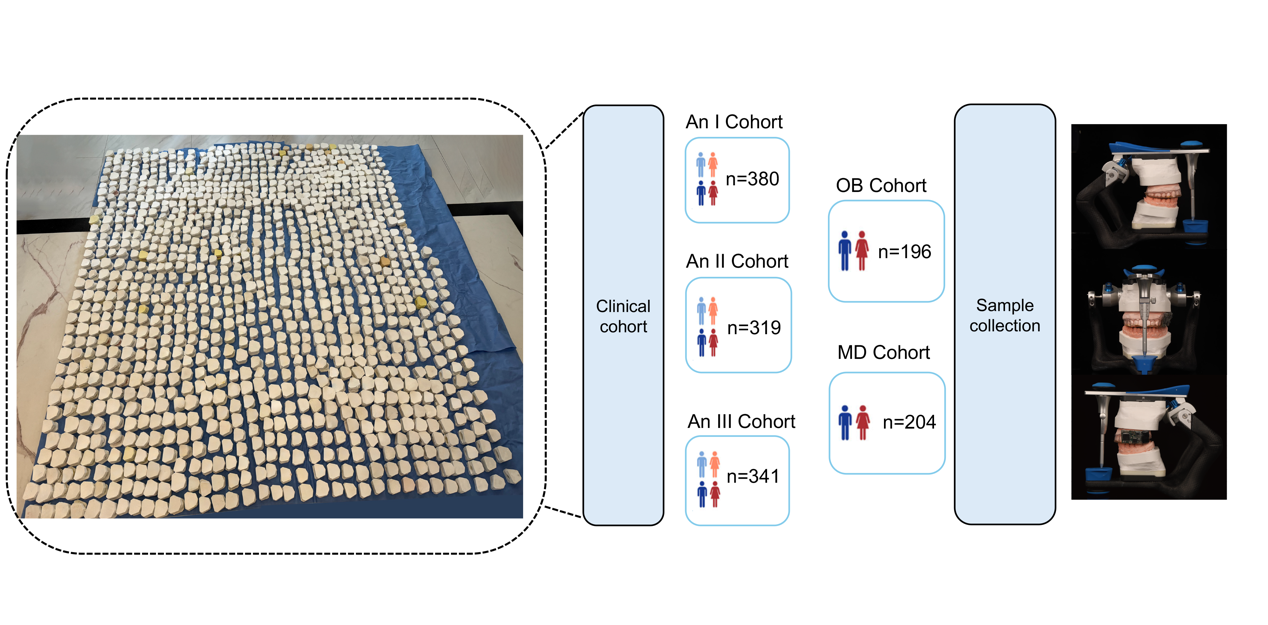
**

**Figure. S13|** **Process of collecting malocclusion data using ARIA.**

**
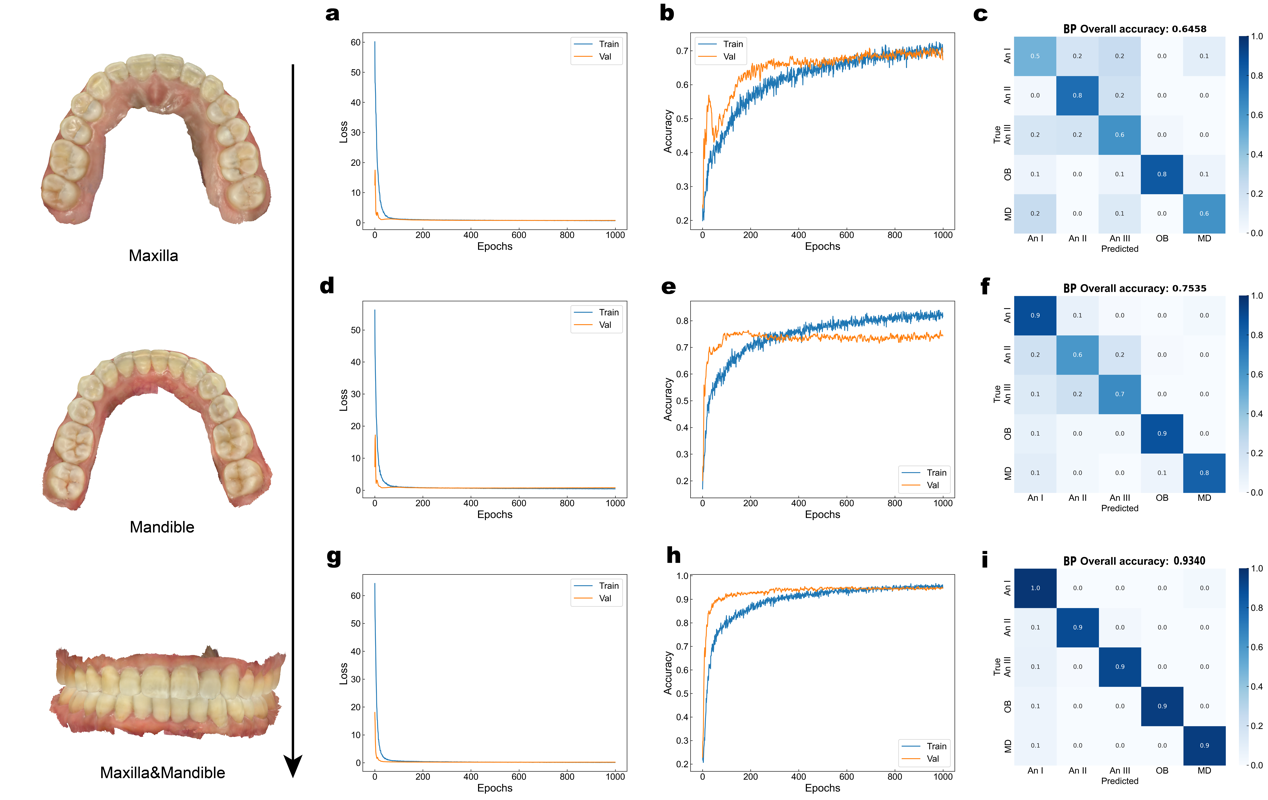
**

**Figure. S14|** **ML Performance of BP neural network algorithm in malocclusion classification. a.** Normalized loss of the training and testing data based on maxillar data.**b.** Normalized accuracy during 100-epoch iterations for the training and testing data based on maxillar data. **c**. Confusion matrix displaying the classification accuracy for predicting each type malocclusion based on maxillar data. **d.** Normalized loss of the training and testing data based on mandibular data.**e.** Normalized accuracy during 100-epoch iterations for the training and testing data based on mandibular data. **f**. Confusion matrix displaying the classification accuracy for predicting each type malocclusion based on mandibular data. **g.** Normalized loss of the training and testing data based on maxillar and mandibular data.**h.** Normalized accuracy during 100-epoch iterations for the training and testing data based on maxillar and mandibular data. **i**. Confusion matrix displaying the classification accuracy for predicting each type malocclusion based on maxillar and mandibular data.


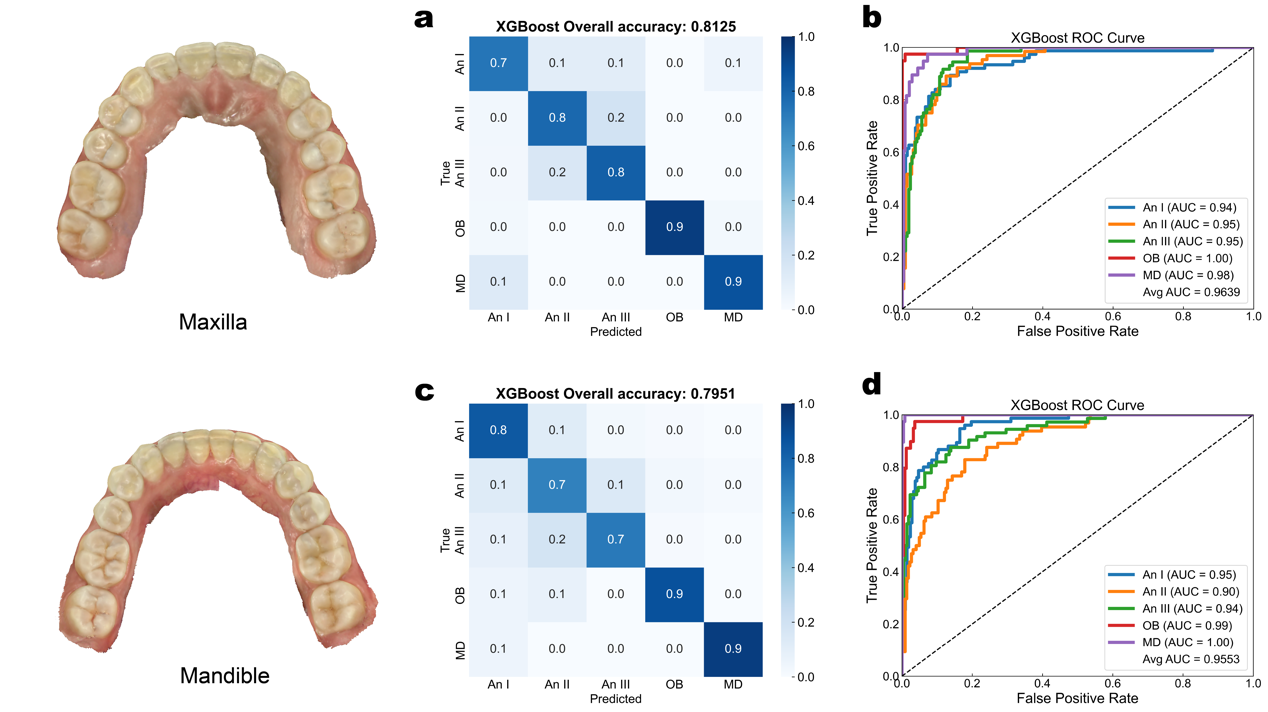


**Figure. S15|** **ML Performance of XGboost algorithm in malocclusion classification. a.** The confusion matrix shows the classification accuracy based on the maxillary data. **b**. Receiver Operating Characteristic Curve (ROC curve) of XGboost based on the maxillary data. **c**. The confusion matrix shows the classification accuracy based on the mandibular data. **d**. Receiver Operating Characteristic Curve (ROC curve) of XGboost based on the mandibular data.

**
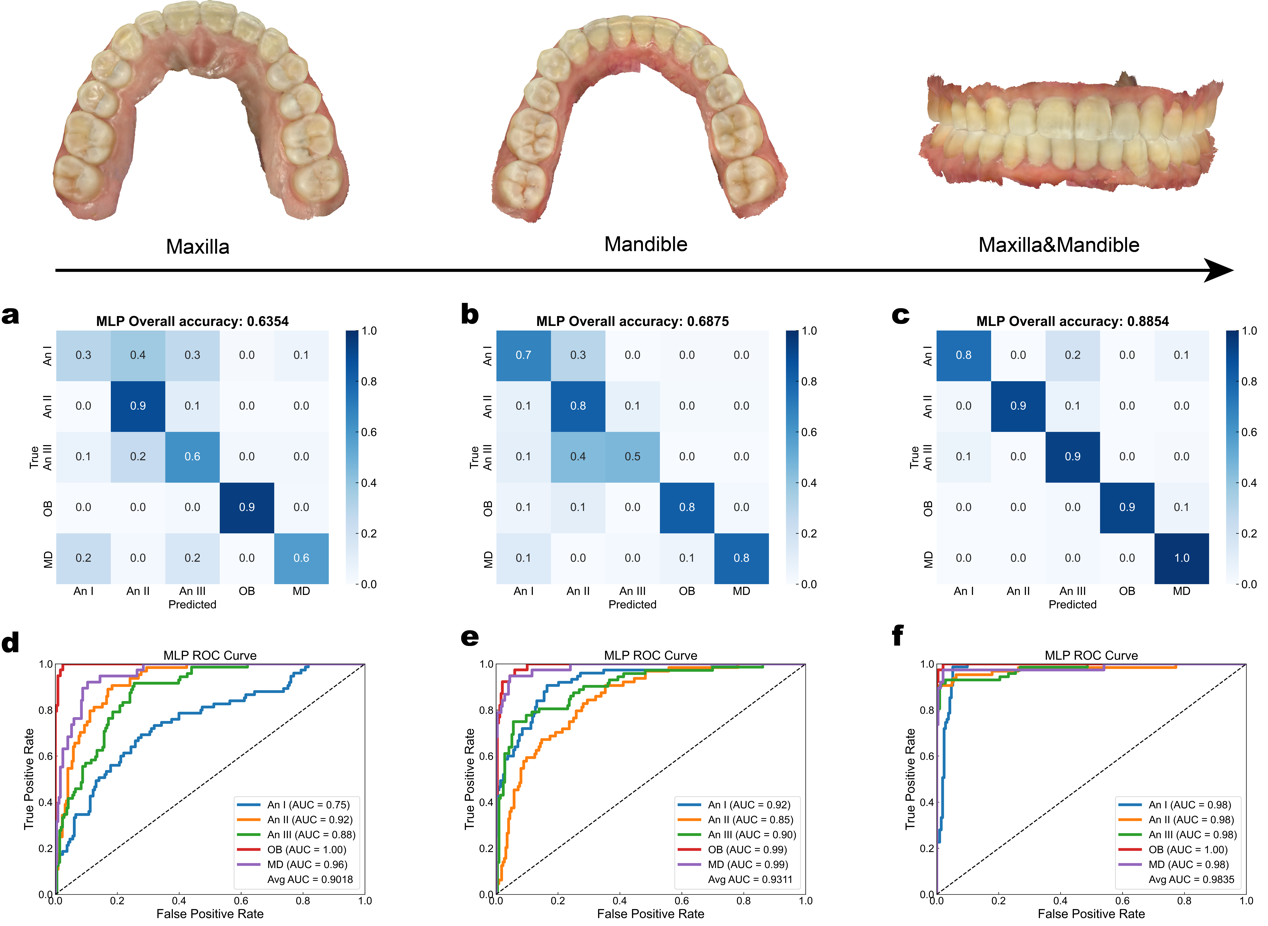
**

**Figure. S16|** **ML Performance of MLP algorithm in malocclusion classification. a.** The confusion matrix shows the classification accuracy based on the maxillary data. **b**. The confusion matrix shows the classification accuracy based on the mandibular data. **c**. The confusion matrix shows the classification accuracy based on the maxillary and mandibular data. **d**. Receiver Operating Characteristic Curve (ROC curve) of MLP based on the maxillary data. **e**. Receiver Operating Characteristic Curve (ROC curve) of MLP based on the mandibular data. **f**. Receiver Operating Characteristic Curve (ROC curve) of MLP based on the maxillary and mandibular data.

**
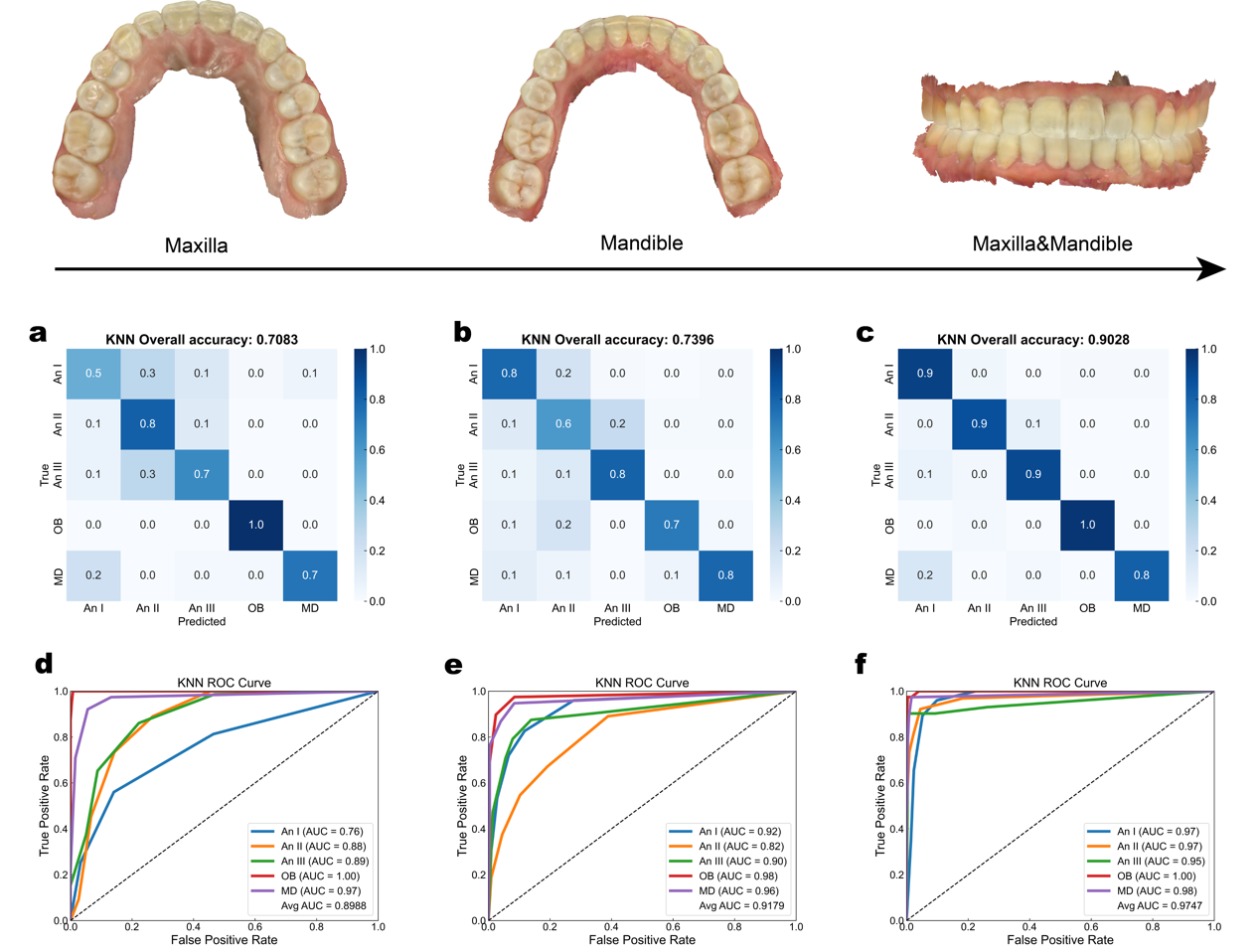
**

**Figure. S17|** **ML Performance of KNN algorithm in malocclusion classification. a.** The confusion matrix shows the classification accuracy based on the maxillary data. **b**. The confusion matrix shows the classification accuracy based on the mandibular data. **c**. The confusion matrix shows the classification accuracy based on the maxillary and mandibular data. **d**. Receiver Operating Characteristic Curve (ROC curve) of KNN based on the maxillary data. **e**. Receiver Operating Characteristic Curve (ROC curve) of KNN based on the mandibular data. **f**. Receiver Operating Characteristic Curve (ROC curve) of KNN based on the maxillary and mandibular data.

**
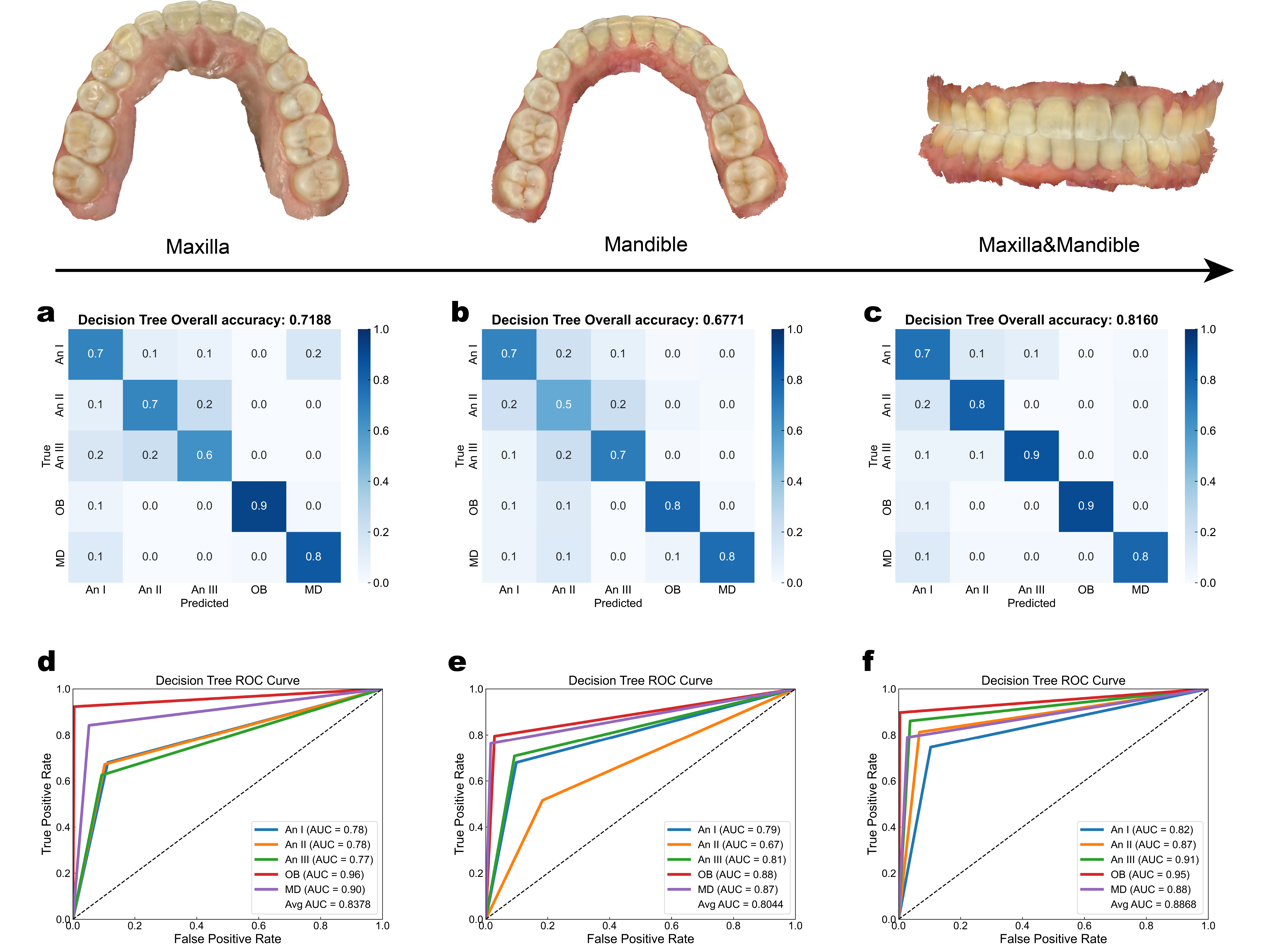
**

**Figure. S18|** **ML Performance of Decision tree algorithm in malocclusion classification. a.** The confusion matrix shows the classification accuracy based on the maxillary data. **b**. The confusion matrix shows the classification accuracy based on the mandibular data. **c**. The confusion matrix shows the classification accuracy based on the maxillary and mandibular data. **d**. Receiver Operating Characteristic Curve (ROC curve) of Decision tree based on the maxillary data. **e**. Receiver Operating Characteristic Curve (ROC curve) of Decision tree based on the mandibular data. **f**. Receiver Operating Characteristic Curve (ROC curve) of Decision tree based on the maxillary and mandibular data.

**
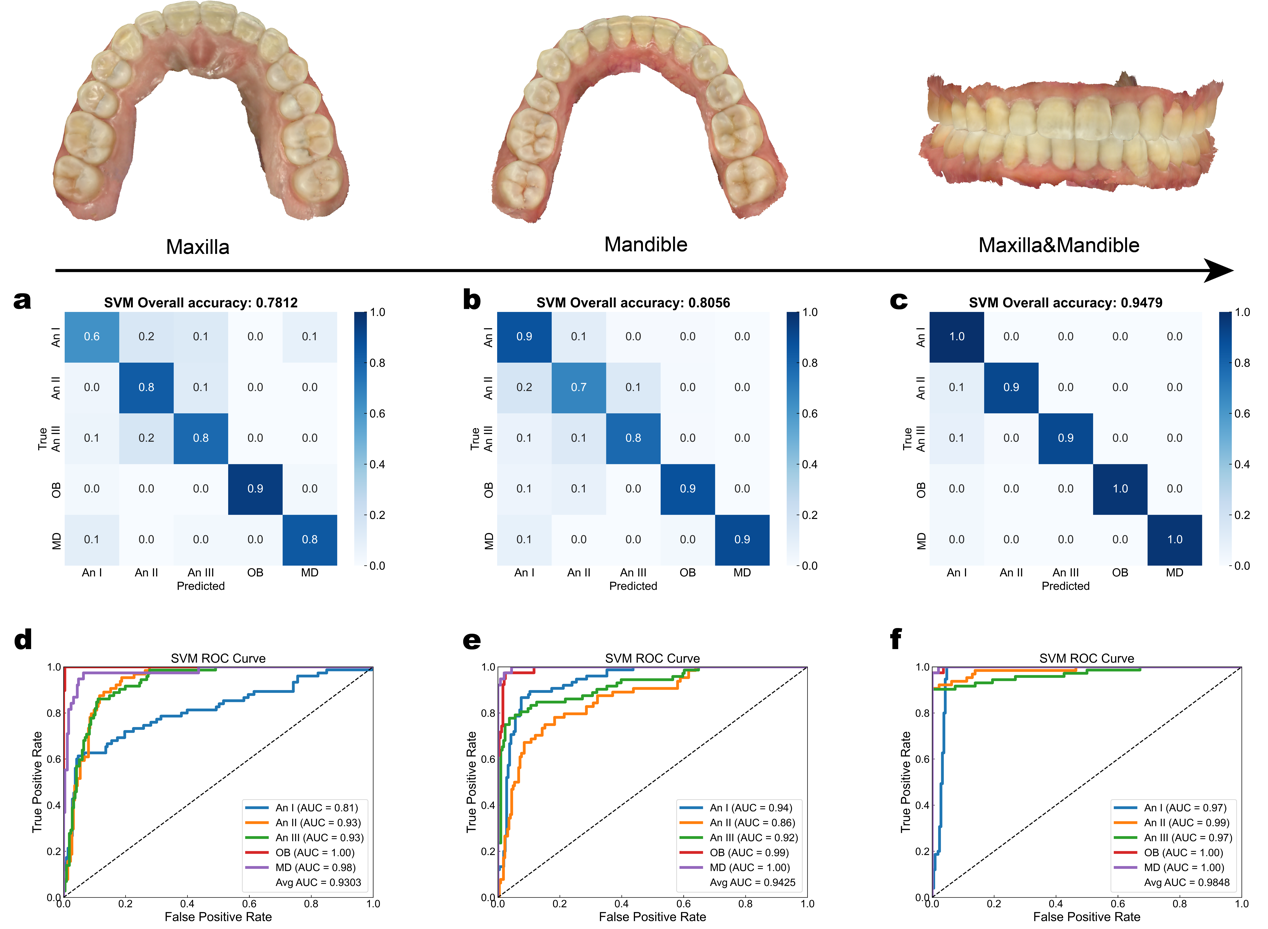
**

**Figure. S19|** **ML Performance of SVM algorithm in malocclusion classification. a.** The confusion matrix shows the classification accuracy based on the maxillary data. **b**. The confusion matrix shows the classification accuracy based on the mandibular data. **c**. The confusion matrix shows the classification accuracy based on the maxillary and mandibular data. **d**. Receiver Operating Characteristic Curve (ROC curve) of SVM based on the maxillary data. **e**. Receiver Operating Characteristic Curve (ROC curve) of SVM based on the mandibular data. **f**. Receiver Operating Characteristic Curve (ROC curve) of SVM based on the maxillary and mandibular data.

**
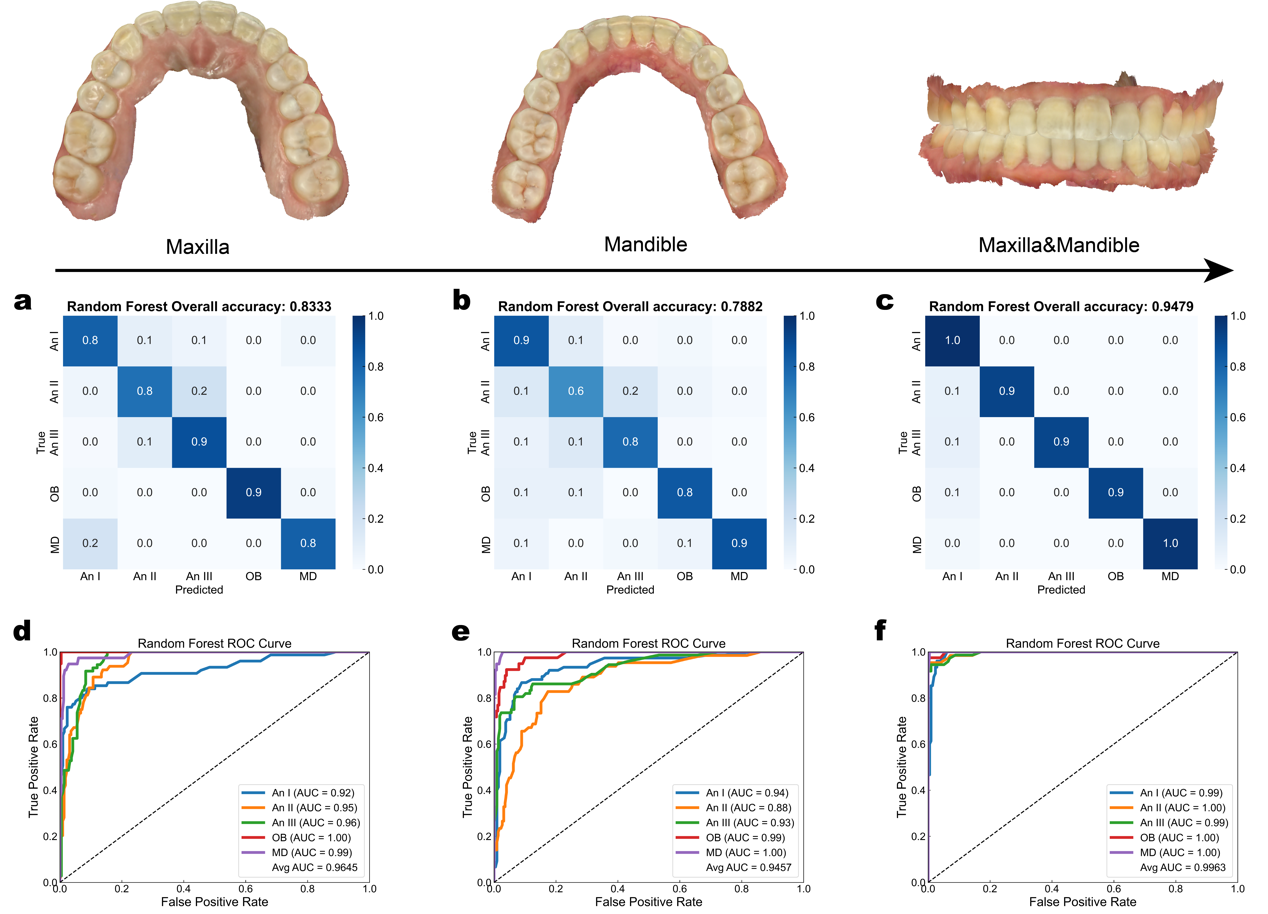
**

**Figure. S20|** **ML Performance of Random Forest algorithm in malocclusion classification. a.** The confusion matrix shows the classification accuracy based on the maxillary data. **b**. The confusion matrix shows the classification accuracy based on the mandibular data. **c**. The confusion matrix shows the classification accuracy based on the maxillary and mandibular data. **d**. Receiver Operating Characteristic Curve (ROC curve) of Random Forest based on the maxillary data. **e**. Receiver Operating Characteristic Curve (ROC curve) of Random Forest based on the mandibular data. **f**. Receiver Operating Characteristic Curve (ROC curve) of Random Forest based on the maxillary and mandibular data.

**
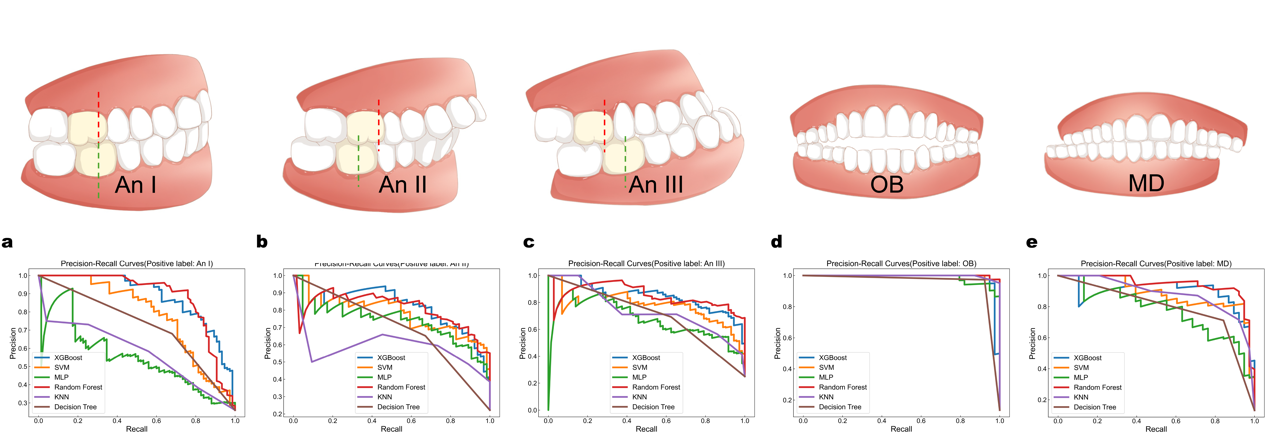
**

**Figure. S21|** **Comparison of ML algorithms based on maxillary data.** **a**. Precision–recall curve of different ML models for Angle class I malocclusion diagnosis. **b**. Precision–recall curve of different ML models for Angle class II malocclusion diagnosis. **c**. Precision–recall curve of different ML models for Angle class III malocclusion diagnosis. **d**. Precision–recall curve of different ML models for open bite malocclusion diagnosis. **e**. Precision–recall curve of different ML models for mandibular deviation diagnosis.

**
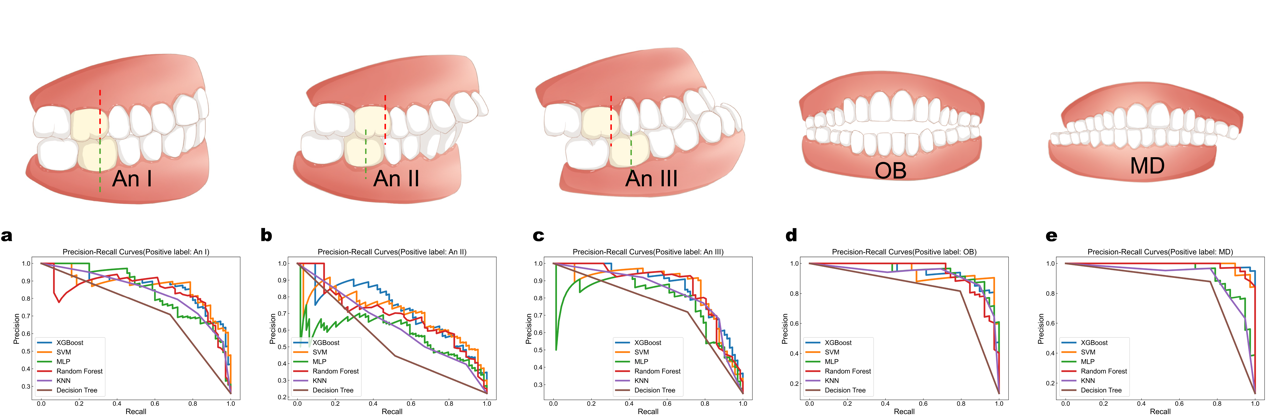
**

**Figure. S22|** **Comparison of ML algorithms based on mandibular data a**. Precision–recall curve of different ML models for Angle class I malocclusion diagnosis. **b**. Precision–recall curve of different ML models for Angle class II malocclusion diagnosis. **c**. Precision–recall curve of different ML models for Angle class III malocclusion diagnosis. **d**. Precision–recall curve of different ML models for open bite malocclusion diagnosis. **e**. Precision–recall curve of different ML models for mandibular deviation diagnosis.

**
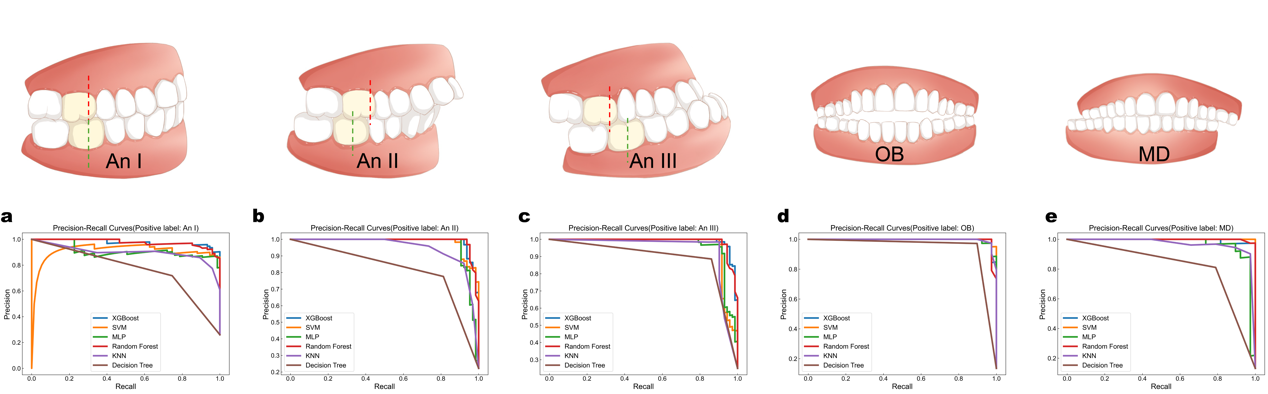
**

**Figure. S23|** **Comparison of ML algorithms based on maxillary and mandibular data a**. Precision–recall curve of different ML models for Angle class I malocclusion diagnosis. **b**. Precision–recall curve of different ML models for Angle class II malocclusion diagnosis. **c**. Precision–recall curve of different ML models for Angle class III malocclusion diagnosis. **d**. Precision–recall curve of different ML models for open bite malocclusion diagnosis. **e**. Precision–recall curve of different ML models for mandibular deviation diagnosis.

**
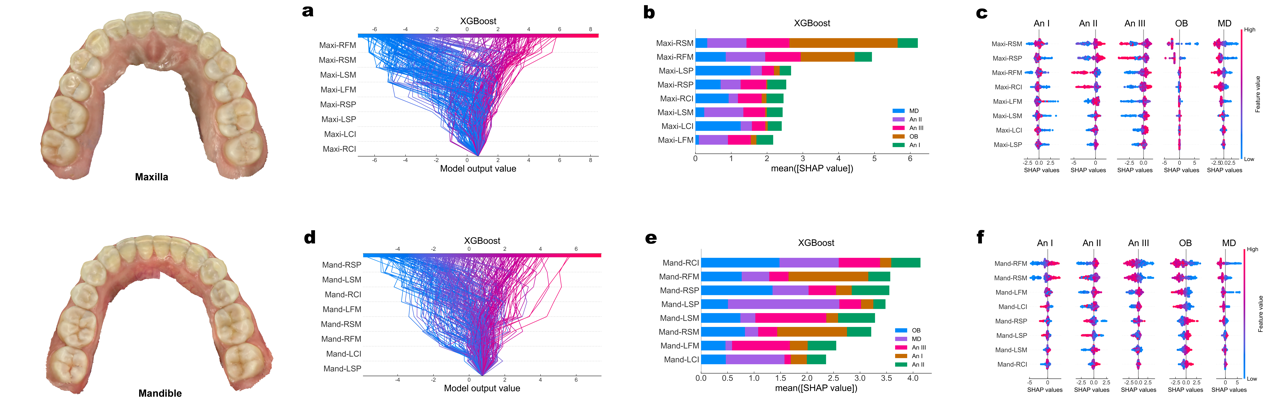
**

**Figure. S24|** **ML prediction performance based on XGBoost on malocclusion classification based on data from maxilla and mandible respectively.** (a) SHAP decision plot explaining how a XGBoost model, based on data from maxilla, arrives at each malocclusion classification for every data point using both physiological and chemical features. Each decision line tracks the features contributions to every individual classification. (b) Stacked bar plot of feature importance showing their contribution to each malocclusion type based on data from the maxilla. (c) Shapley additive explanation (SHAP) summary plot with respect to a XGBoost model based on the dataset collected by ARIA. (d) SHAP decision plot explaining how a XGBoost model, based on data from mandible, arrives at each malocclusion classification for every data point using both physiological and chemical features. Each decision line tracks the features contributions to every individual classification. (e) Stacked bar plot of feature importance showing their contribution to each malocclusion type based on data from the mandible. (f) Shapley additive explanation (SHAP) summary plot with respect to a XGBoost model based on the dataset collected by ARIA.


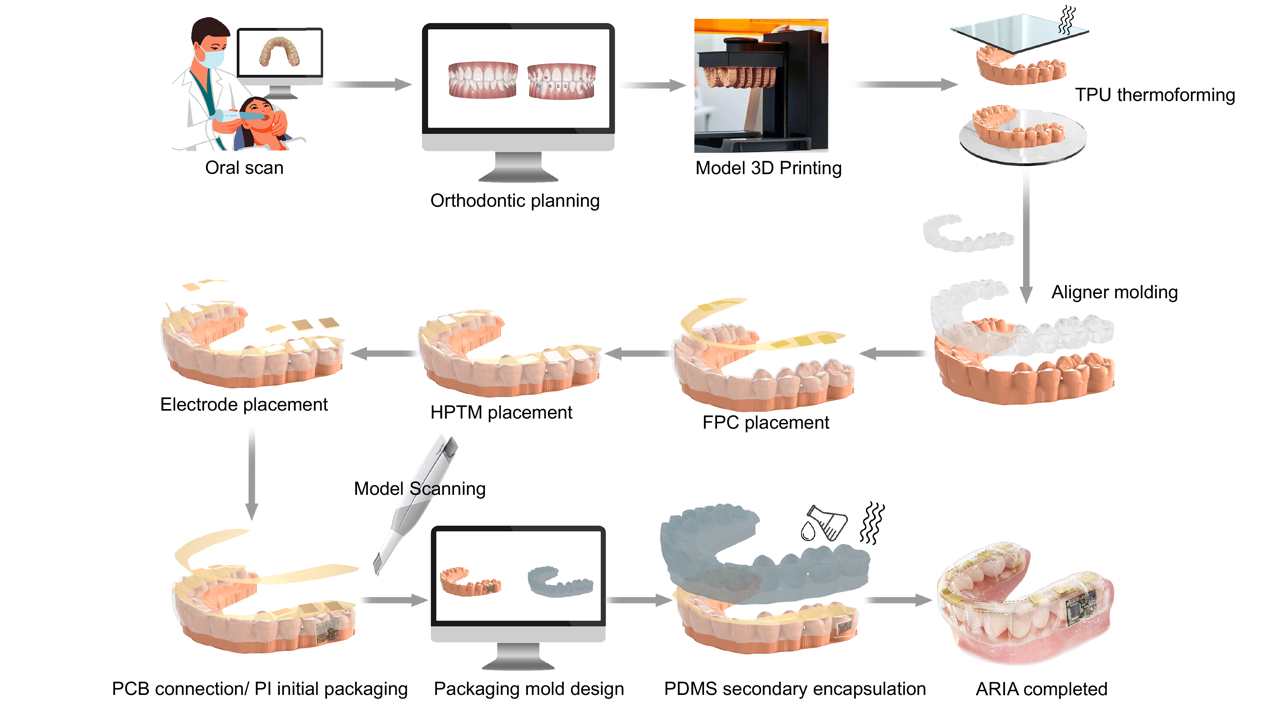


**Figure. S25|** **Fabrication process of the ARIA.**

Supplementary References

[1] H. Pei, J. Jing, Y. Chen, J. Guo, N. Chen, 3D printing of PVDF-based piezoelectric nanogenerator from programmable metamaterial design: Promising strategy for flexible electronic skin, Nano Energy 109 (2023) 108303.

[2] N.A. Shepelin, P.C. Sherrell, E.N. Skountzos, E. Goudeli, J. Zhang, V.C. Lussini, B. Imtiaz, K.A.S. Usman, G.W. Dicinoski, J.G. Shapter, J.M. Razal, A.V. Ellis, Interfacial piezoelectric polarization locking in printable Ti3C2Tx MXene-fluoropolymer composites, Nature Communications 12(1) (2021) 3171.

[3] I. Katsouras, K. Asadi, M. Li, T.B. van Driel, K.S. Kjær, D. Zhao, T. Lenz, Y. Gu, P.W.M. Blom, D. Damjanovic, M.M. Nielsen, D.M. de Leeuw, The negative piezoelectric effect of the ferroelectric polymer poly(vinylidene fluoride), Nature Materials 15(1) (2016) 78-84.

[4] X. Yuan, A. Yan, Z. Lai, Z. Liu, Z. Yu, Z. Li, Y. Cao, S. Dong, A poling-free PVDF nanocomposite via mechanically directional stress field for self-powered pressure sensor application, Nano Energy 98 (2022) 107340.

[5] A. Baji, Y.-W. Mai, Q. Li, Y. Liu, Nanoscale investigation of ferroelectric properties in electrospun barium titanate/polyvinylidene fluoride composite fibers using piezoresponse force microscopy, Composites Science and Technology 71(11) (2011) 1435-1440.

[6] H.-S. Wu, S.-M. Wei, S.-W. Chen, H.-C. Pan, W.-P. Pan, S.-M. Huang, M.-L. Tsai, P.-K. Yang, Metal-Free Perovskite Piezoelectric Nanogenerators for Human–Machine Interfaces and Self-Powered Electrical Stimulation Applications, Advanced Science 9(18) (2022) 2105974.

[7] P. Sharma, D. Wu, S. Poddar, T.J. Reece, S. Ducharme, A. Gruverman, Orientational imaging in polar polymers by piezoresponse force microscopy, Journal of Applied Physics 110(5) (2011) 052010.

[8] J. Tao, Y. Chen, A. Bhardwaj, L. Wen, J. Li, O.V. Kolosov, Y. Lin, Z. Hong, Z. Huang, S. Mathur, Combating Li metal deposits in all-solid-state battery via the piezoelectric and ferroelectric effects, Proceedings of the National Academy of Sciences 119(41) (2022) e2211059119.

[9] P.-K. Yang, S.-A. Chou, C.-H. Hsu, R.J. Mathew, K.-H. Chiang, J.-Y. Yang, Y.-T. Chen, Tin disulfide piezoelectric nanogenerators for biomechanical energy harvesting and intelligent human-robot interface applications, Nano Energy 75 (2020) 104879.

[10] Y. Lu, H. Tian, J. Cheng, F. Zhu, B. Liu, S. Wei, L. Ji, Z.L. Wang, Decoding lip language using triboelectric sensors with deep learning, Nature Communications 13(1) (2022) 1401.

[11] W. Fan, R. Lei, H. Dou, Z. Wu, L. Lu, S. Wang, X. Liu, W. Chen, M. Rezakazemi, T.M. Aminabhavi, Y. Li, S. Ge, Sweat permeable and ultrahigh strength 3D PVDF piezoelectric nanoyarn fabric strain sensor, Nature Communications 15(1) (2024) 3509.

[12] J. Zhang, T. Yang, G. Tian, B. Lan, W. Deng, L. Tang, Y. Ao, Y. Sun, W. Zeng, X. Ren, Z. Li, L. Jin, W. Yang, Spatially Confined MXene/PVDF Nanofiber Piezoelectric Electronics, Advanced Fiber Materials 6(1) (2024) 133-144.

[13] R. Guo, Y. Fang, Z. Wang, A. Libanori, X. Xiao, D. Wan, X. Cui, S. Sang, W. Zhang, H. Zhang, J. Chen, Deep Learning Assisted Body Area Triboelectric Hydrogel Sensor Network for Infant Care, Advanced Functional Materials 32(35) (2022) 2204803.

[14] L. Chen, K. Yuan, S. Chen, Y. Huang, H. Askari, N. Yu, J. Mo, N. Xu, M. Wu, H. Chen, A. Khajepour, Z. Wang, Triboelectric nanogenerator sensors for intelligent steering wheel aiming at automated driving, Nano Energy 113 (2023) 108575.

[15] C. Zheng, W. Li, Y. Shi, S. Wei, K. Liu, J. Cheng, L. Ji, Y. Lu, Stretchable self-adhesive and self-powered smart bandage for motion perception and motion intention recognition, Nano Energy 109 (2023) 108245.

[16] D. Yang, K. Zhao, R. Yang, S.-W. Zhou, M. Chen, H. Tian, D.-H. Qu, A Rational Design of Bio-Derived Disulfide CANs for Wearable Capacitive Pressure Sensor, Advanced Materials n/a(n/a) (2024) 2403880.

[17] J. Yang, D. Tang, J. Ao, T. Ghosh, T.V. Neumann, D. Zhang, Y. Piskarev, T. Yu, V.K. Truong, K. Xie, Y.-C. Lai, Y. Li, M.D. Dickey, Ultrasoft Liquid Metal Elastomer Foams with Positive and Negative Piezopermittivity for Tactile Sensing, Advanced Functional Materials 30(36) (2020) 2002611.

[18] S. Lee, S. Franklin, F.A. Hassani, T. Yokota, M.O.G. Nayeem, Y. Wang, R. Leib, G. Cheng, D.W. Franklin, T. Someya, Nanomesh pressure sensor for monitoring finger manipulation without sensory interference, Science 370(6519) (2020) 966-970.

[19] X. Lin, Y. Teng, H. Xue, Y. Bing, F. Li, J. Wang, J. Li, H. Zhao, T. Zhang, Janus Conductive Mechanism: An Innovative Strategy Enabling Ultra-Wide Linearity Range Pressure Sensing for Multi-Scenario Applications, Advanced Functional Materials n/a(n/a) (2024) 2316314.

[20] H. Cheng, B. Wang, K. Yang, Y.Q. Yang, C. Wang, A high-performance piezoresistive sensor based on poly (styrene-co-methacrylic acid)@polypyrrole microspheres/graphene-decorated TPU electrospun membrane for human motion detection, Chemical Engineering Journal 426 (2021) 131152.

[21] Y.S. Fang, Y.J. Zou, J. Xu, G.R. Chen, Y.H. Zhou, W.L. Deng, X. Zhao, M. Roustaei, T.K. Hsiai, J. Chen, Ambulatory Cardiovascular Monitoring Via a Machine-Learning-Assisted Textile Triboelectric Sensor, Advanced Materials 33(41) (2021).

[22] Z.W. Lin, G.Q. Zhang, X. Xiao, C. Au, Y.H. Zhou, C.C. Sun, Z.H. Zhou, R. Yan, E.D. Fan, S.B. Si, L. Weng, S. Mathur, J. Yang, J. Chen, A Personalized Acoustic Interface for Wearable Human-Machine Interaction, Advanced Functional Materials 32(9) (2022).

[23] Y. Long, P.S. He, R.X. Xu, T. Hayasaka, Z.C. Shao, J.W. Zhong, L.W. Lin, Molybdenum-carbide-graphene composites for paper-based strain and acoustic pressure sensors, Carbon 157 (2020) 594-601.

[24] H.J. Lee, J.C. Yang, J. Choi, J. Kim, G.S. Lee, S.P. Sasikala, G.H. Lee, S.H.K. Park, H.M. Lee, J.Y. Sim, S. Park, S.O. Kim, Hetero-Dimensional 2D Ti<sub>3</sub>C<sub>2</sub>T<i><sub>x</sub></i> MXene and 1D Graphene Nanoribbon Hybrids for Machine Learning-Assisted Pressure Sensors, Acs Nano 15(6) (2021) 10347-10356.

[25] Q.F. Shi, Z.X. Zhang, T.Y.Y. He, Z.D. Sun, B.J. Wang, Y.Q. Feng, X.C. Shan, B. Salam, C. Lee, Deep learning enabled smart mats as a scalable floor monitoring system, Nature Communications 11(1) (2020).

[26] P. Tan, X. Han, Y. Zou, X. Qu, J. Xue, T. Li, Y. Wang, R. Luo, X. Cui, Y. Xi, L. Wu, B. Xue, D. Luo, Y. Fan, X. Chen, Z. Li, Z.L. Wang, Self-Powered Gesture Recognition Wristband Enabled by Machine Learning for Full Keyboard and Multicommand Input, Advanced Materials 34(21) (2022) 2200793.

[27] H.S. Wang, S.K. Hong, J.H. Han, Y.H. Jung, H.K. Jeong, T.H. Im, C.K. Jeong, B.Y. Lee, G. Kim, C.D. Yoo, K.J. Lee, Biomimetic and flexible piezoelectric mobile acoustic sensors with multiresonant ultrathin structures for machine learning biometrics, Sci. Adv. 7(7) (2021).

[28] H. Jeong, J.Y. Yoo, W. Ouyang, A. Greane, A.J. Wiebe, I. Huang, Y.J. Lee, J.Y. Lee, J. Kim, X.C. Ni, S. Kim, H.L. Huynh, I. Zhong, Y.X. Chin, J.Y. Gu, A.M. Johnson, T. Brancaccio, J.A. Rogers, Closed-loop network of skin-interfaced wireless devices for quantifying vocal fatigue and providing user feedback, Proceedings of the National Academy of Sciences of the United States of America 120(9) (2023).
